# The endosomal pH regulator NHE9 is a driver of stemness in glioblastoma

**DOI:** 10.1093/pnasnexus/pgac013

**Published:** 2022-03-09

**Authors:** Myungjun Ko, Monish R Makena, Paula Schiapparelli, Paola Suarez-Meade, Allatah X Mekile, Bachchu Lal, Hernando Lopez-Bertoni, Kristen L Kozielski, Jordan J Green, John Laterra, Alfredo Quiñones-Hinojosa, Rajini Rao

**Affiliations:** Department of Physiology, The Johns Hopkins University School of Medicine, Baltimore, MD, 21205, USA; Department of Neurosurgery, Mayo Clinic College of Medicine, Jacksonville, FL, 32224, USA; Department of Physiology, The Johns Hopkins University School of Medicine, Baltimore, MD, 21205, USA; Department of Neurosurgery, Mayo Clinic College of Medicine, Jacksonville, FL, 32224, USA; Department of Neurosurgery, Mayo Clinic College of Medicine, Jacksonville, FL, 32224, USA; Department of Physiology, The Johns Hopkins University School of Medicine, Baltimore, MD, 21205, USA; Hugo W. Moser Research Institute at Kennedy Krieger, Baltimore, MD, USA; Department of Neurology, The Johns Hopkins University School of Medicine, Baltimore, MD, 21205, USA; Hugo W. Moser Research Institute at Kennedy Krieger, Baltimore, MD, USA; Department of Neurology, The Johns Hopkins University School of Medicine, Baltimore, MD, 21205, USA; Department of Biomedical Engineering, The Johns Hopkins University School of Medicine, Baltimore, MD, 21205, USA; Department of Electrical and Computer Engineering, Technical University of Munich, Munich, Germany; Department of Biomedical Engineering, The Johns Hopkins University School of Medicine, Baltimore, MD, 21205, USA; Hugo W. Moser Research Institute at Kennedy Krieger, Baltimore, MD, USA; Department of Neurology, The Johns Hopkins University School of Medicine, Baltimore, MD, 21205, USA; Department of Neurosurgery, Mayo Clinic College of Medicine, Jacksonville, FL, 32224, USA; Department of Physiology, The Johns Hopkins University School of Medicine, Baltimore, MD, 21205, USA

**Keywords:** glioblastoma, Na^+^/H^+^ exchanger, endosomes, receptor tyrosine kinase, STAT3

## Abstract

A small population of self-renewing stem cells initiate tumors and maintain therapeutic resistance in glioblastoma (GBM). Given the limited treatment options and dismal prognosis for this disease, there is urgent need to identify drivers of stem cells that could be druggable targets. Previous work showed that the endosomal pH regulator NHE9 is upregulated in GBM and correlates with worse survival prognosis. Here, we probed for aberrant signaling pathways in patient-derived GBM cells and found that NHE9 increases cell surface expression and phosphorylation of multiple receptor tyrosine kinases (RTKs) by promoting their escape from lysosomal degradation. Downstream of NHE9-mediated receptor activation, oncogenic signaling pathways converged on the JAK2-STAT3 transduction axis to induce pluripotency genes Oct4 and Nanog and suppress markers of glial differentiation. We used both genetic and chemical approaches to query the role of endosomal pH in GBM phenotypes. Loss-of-function mutations in NHE9 that failed to alkalinize endosomal lumen did not increase self-renewal capacity of gliomaspheres in vitro. However, monensin, a chemical mimetic of Na^+^/H^+^ exchanger activity, and the H^+^ pump inhibitor bafilomycin bypassed NHE9 to directly alkalinize the endosomal lumen resulting in stabilization of RTKs and induction of Oct4 and Nanog. Using orthotopic models of primary GBM cells we found that NHE9 increased tumor initiation in vivo. We propose that NHE9 initiates inside-out signaling from the endosomal lumen, distinct from the established effects of cytosolic and extracellular pH on tumorigenesis. Endosomal pH may be an attractive therapeutic target that diminishes stemness in GBM, agnostic of specific receptor subtype.

Significance Statement A well-known hallmark of cancer is excessive acidification of tumor microenvironment, caused by upregulation of Na^+^/H^+^ exchanger activity on the cancer cell membrane. However, the role of organellar pH in tumor biology has not been established. This study identifies a mechanistic link between upregulation of the endosomal Na^+^/H^+^ exchanger NHE9 and stemness properties in GBM, the most malignant and common brain tumor in adults. By increasing pH of the recycling endosome, NHE9 exerts a broad effect on post-translational stability and activation of multiple RTKs, leading to increased stem cell-like properties of self-renewal and tumor initiation in GBM models. Our findings suggest that targeting NHE9 or endosomal pH could be an effective strategy for receptor agnostic GBM treatment.

## Introduction

Glioblastoma (GBM) is the most frequent type of brain tumor in adults and the most malignant form (grade IV) of glioma with average 3-year survival rate of less than 10% (1–3). The currently available “gold standard” for GBM patients is surgical resection of the tumor mass, followed by radiotherapy and concurrent chemotherapy with the alkylating agent temozolomide (TMZ) (4–6). Median survival with TMZ treatment and radiotherapy combined after surgical intervention has only increased marginally from 12 to 14.6 months (3, 7). The problem is that there are no other chemotherapeutic drugs or targets that have been approved and effective for GBM patients. Thus, a global effort in the brain tumor field has been focused on finding novel therapeutic targets for this rapidly fatal brain tumor. Of central importance in these investigations are cancer stem cells, a subset of cancer cells with stem cell-like characteristics, that may be responsible for the resistance of GBM to conventional therapies and recurrence of the disease (8, 9). There is a high unmet need to find novel therapeutic targets that are drivers of cancer stem cells.

Aberrant signaling pathways and epigenetic changes are the main drivers of GBM tumor phenotype and resistance to therapy. Receptor tyrosine kinases (RTKs) regulate tumor cell proliferation, migration, differentiation, and survival through downstream signaling pathways. Although expression and activation of RTKs is frequently aberrant in GBM, redundant signaling pathways and tumor heterogeneity in RTK expression make it extremely hard for RTK-targeted therapy to be effective (10, 11). Combination therapy—targeting multiple RTKs, has been suggested (12) but the plasticity of tumor cells and their stochastic response has prevented this approach from being effective and approved for patients. Furthermore, a small population of stem cells in GBM confer treatment resistance and self-renewal (8, 13). Thus, a druggable target that controls both stemness characteristics and pan-receptor clearance in GBM cells could circumvent shortcomings in current therapy.

A common pathophysiological hallmark of tumors is a perturbation in pH dynamics (14–16). The accumulation of acidic byproducts of metabolism together with poor vascularization and accompanying hypoxia demands net acid extrusion from the cancer cell, which drives tumor microenvironment to pH 6.5 or lower. A well-studied mechanism underlying these pH changes is activation of the plasma membrane Na^+^/H^+^ exchanger NHE1, which plays a central role in tumor cell migration and invasion (17, 18). In contrast, several poorly understood intracellular NHE isoforms (19) that regulate the lumenal pH of endosomes, Golgi and *trans*-Golgi network are emerging as potential drivers of tumorigenesis and drug resistance, and offer exciting new mechanistic insights that could be therapeutically exploited (20–22).

One such intracellular isoform is NHE9, which localizes to recycling endosomes and is widely expressed in the brain (23, 24). Even small changes in Na^+^/H^+^ exchange activity can cause large shifts in pH within the limited confines of the endosomal lumen (20, 24). NHE9 has been implicated in multiple malignancies, including GBM, prostate cancer, colorectal cancer, esophageal squamous carcinoma, oral cancer, and ovarian cancer (reviewed in (25)). However, the underlying mechanistic basis for these observations is largely unclear.

In this study, we sought to broaden our initial observation that NHE9 increases membrane persistence of the epidermal growth factor receptor (EGFR) in GBM cells (20). Our new findings suggest that NHE9 has a more global effect on receptor stabilization that is agnostic of GBM subtype. We found JAK2/STAT3 serves as a common signal transduction pathway downstream of NHE9-mediated RTK activation. STAT3 is a critical signaling molecule (26–28) that controls a small population of self-renewing stem cells that occupy the apex of the tumor cell hierarchy and drive initiation, growth, and therapeutic resistance in GBM. These observations led us to define the intermediate steps linking NHE9 to self-renewal capacity in gliomaspheres in vitro, and tumor initiation in vivo. Both genetic and pharmacological tools point toward a causal role for endosomal pH in GBM stem cell-like properties. In summary, we report that “inside-out” signaling from the endosomal lumen can drive stemness, which could explain poor survival prognosis GBM patients with elevated NHE9 expression. We suggest that inhibition of NHE9 will attenuate oncogenic signaling in a receptor-agnostic manner, opening potential avenues for cancer therapy.

## Results

### NHE9 stabilizes multiple RTKs

**Fig. 1.**
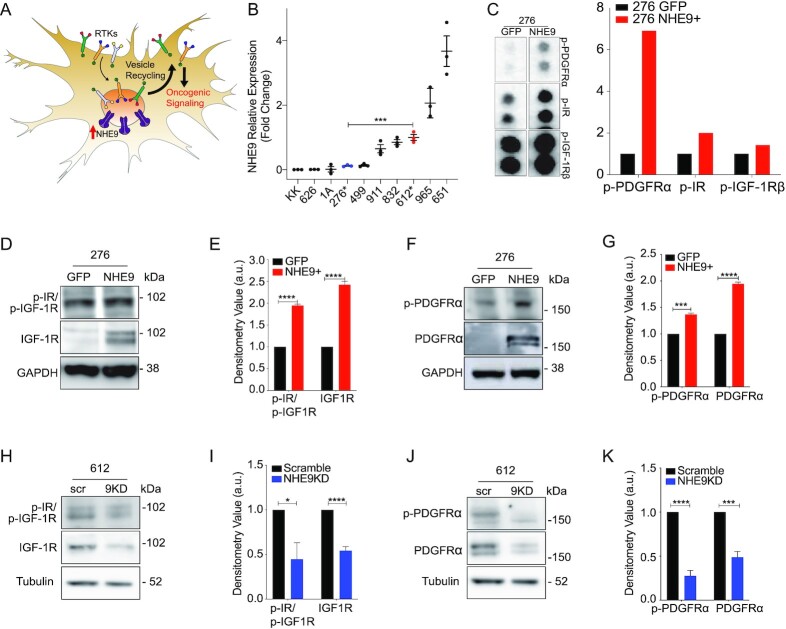
NHE9 increases activation and expression of RTKs. (A) Schematic of hypothesis linking endosomal NHE9 to RTK (RTK) recycling and oncogenic signaling. (B) Relative mRNA expression of NHE9 measured by qPCR for 10 GBM cell lines established from samples obtained from Johns Hopkins neurosurgery operating room. GBM 276 and GBM 612 were chosen to represent low and high expressors, without being outliers. (C) Portion of phospho-RTK dot blot (left) probed with lysates from GBM 276 ectopically expressing GFP or NHE9-GFP. Densitometry quantification (in arbitrary units) of p-PDGFRα, p-IGF-1Rβ, and p-IR from the dot blot (right). (D) and (F) Representative western blot showing p-IGF-1R, p-IR, and p-PDGFRα (top row) and IGF-1R, IR, and PDGFRα (bottom row) for GBM 276 GFP or NHE9-GFP and densitometry quantification (E) and (G). (H) and (J) Representative western blot showing p-IGF-1R, p-IR, and p-PDGFRα (top row) and IGF-1R, IR, and PDGFRα (bottom row) for GBM 612 scramble or shNHE9 and densitometry quantification (I) and (K). Averages are from 3 independently generated transfections.

Multiple steps in the internalization, recycling, and degradation of ligand–receptor complexes are exquisitely dependent on the acidic pH of the endolysosomal lumen, including dissociation of ligand–receptor complex, cargo sorting decisions, and vesicle docking and fusion. Therefore, we hypothesized that dysregulation of pH in the recycling endosome in GBM cells globally elevates surface expression of RTK, leading to prolonged oncogenic signaling (Fig. 1A). A precise molecular tool to constitutively and selectively manipulate the pH of endosomes is by regulating NHE9 activity and expression. Therefore, to test our hypothesis, we screened for NHE9 expression a diverse panel of 10 patient-derived GBM cell lines, established from samples obtained from the operative room at the Johns Hopkins Hospital and propagated in serum-free medium (Fig. 1B). GBM 276 and GBM 612 represent low and high expression of endogenous NHE9, without being outliers, and also represent distinct GBM subtypes—proneural and mesenchymal, respectively (20). Previously, we showed that endosomal pH correlated with NHE9 expression (20), being lower in GBM 276 (5.57 ± 0.13) relative to GBM 612 (6.88 ± 0.13). Isogenic matched pairs from these cells representing loss or gain of NHE9 function were generated, using lentiviral shRNA for NHE9 knockdown (KD) in GBM 612 and transgenic expression of NHE9 tagged with GFP in GBM 276 (Figure S1A–C, Supplementary Material). To ensure that transgene expression of NHE9-GFP physiologically mimics the endogenous high levels of NHE9 in GBM, we demonstrated that endosomal localization of NHE9-GFP in GBM 276 was accompanied by increase in lumen pH to 6.61 ± 0.21, similar to GBM 612. Notably, no change in cytosolic pH was observed in response to manipulation of endosomal NHE expression (20, 29). Thus, these cells are well-suited for mechanistic studies of NHE9 in GBM.

Transgene expression of NHE9 in GBM 276 resulted in the activation of multiple receptors detected on a phospho-RTK dot blot overlaid with GBM cell lysate (Figure S1D, Supplementary Material). The most prominent of these include insulin-like growth factor receptor 1 (IGF-1R), insulin receptor (IR), and platelet-derived growth factor receptor alpha (PDGFRα), (Fig. 1C). Western blot analysis of lysates from GBM 276 cells confirmed that gain of function in NHE9 increased phosphorylation in these receptors (Fig. 1D–G, top panels). Conversely, these receptors showed diminished phosphorylation in response to NHE9 KD in GBM 612 (Fig. 1H–K, top panels). Gain or loss of NHE9 also caused corresponding increase or decrease in total levels RTK proteins (Fig. 1D–K, bottom panels). Notably, transcript levels of these receptors remained unchanged by the expression of NHE9, as determined by qPCR (Figure S1E–G, Supplementary Material) pointing to post-translational mechanisms underlying the differences in receptor levels. Therefore, we hypothesized that NHE9 altered receptor stability and turnover.

**Fig. 2.**
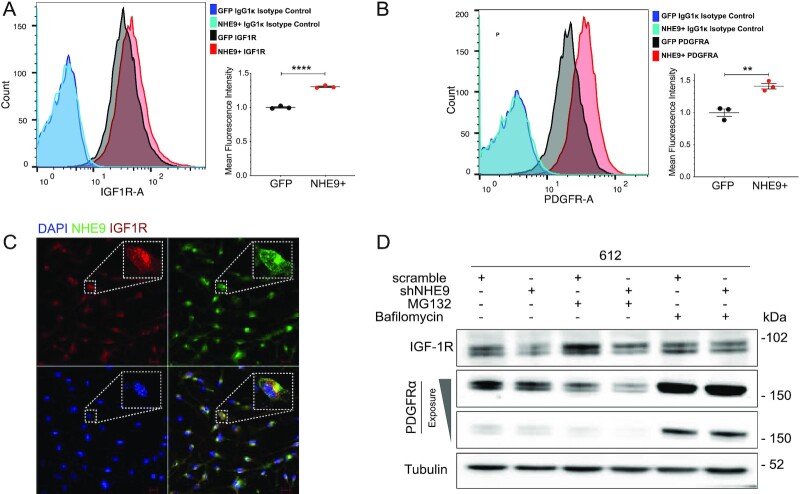
NHE9 increases surface expression and decreases lysosomal degradation of RTKs. (A) and (B) Representative flow cytometry analysis of nonpermeabilized GBM 276 cells expressing GFP or NHE9-GFP, treated with monoclonal antibodies for IGF-1R (A) and PDGFRα (B) in comparison with IgG1 kappa isotope control. Mean fluorescence intensity of peak areas are from triplicates of independently transfected cultures. (C) Immunofluorescence images of GBM 276 cells showing IGF-1R (Red), NHE9-GFP (Green), and DAPI (blue). Merge of all 3 labels shows co-localization of IGF-1R and NHE9 (yellow). Scale bar: 10 µm. (D) Western blotting analysis of IGF-1R and PDGFRα in GBM 612 scramble or NHE9KD cells treated with 25 nM MG132 or 25 nM bafilomycin for 6 h. Note that only bafilomycin diminishes the effect of shNHE9 on receptor levels.

To probe cell surface expression of RTKs, we used flow cytometry analysis of nonpermeabilized GBM 276 cells labeled with antibody against external receptor epitopes. We observed significant right shift of IGF-1R (Fig. 2A) or PDGFRα (Fig. 2B) indicating increased surface expression in response to ectopic expression of NHE9, consistent with our model (Fig. 1A). Immunofluorescence imaging revealed co-localization of IGF-1R with NHE9-GFP in GBM 276 cells (Fig. 2C), reinforcing our hypothesis. Next, we treated cells with MG132 and bafilomycin, to block proteasomal and lysosomal degradation, respectively. In control GBM 612 cells, KD of NHE9 decreased levels of IGF-1R and PDGFRα, similar to results shown in Fig. 1. Whereas MG132 had no effect on RTK levels, treatment with bafilomycin was effective in protecting IGF-1R and PDGFRα protein levels in NHE9 KD cells (Fig. 2D). These observations confirm and extend our earlier findings on EGFR persistence in GBM (20), and serve as a necessary starting point to determine the downstream oncogenic consequences of NHE9 expression.

### NHE9 induces phosphorylation of STAT3, a key prognostic marker in GBM

**Fig. 3.**
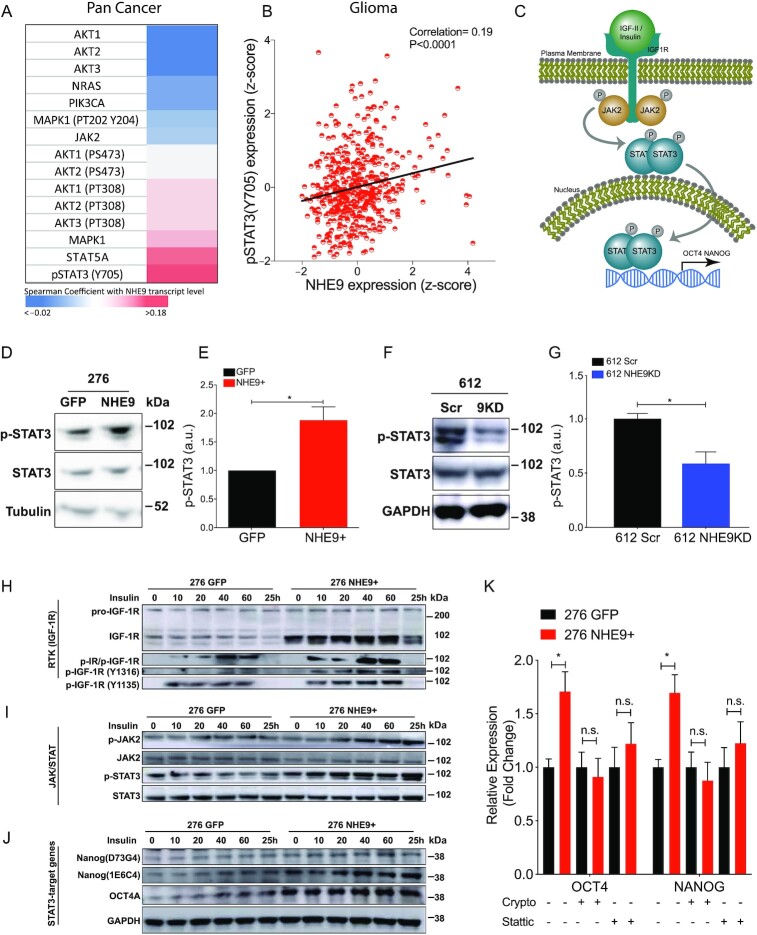
NHE9 activates the STAT3 signaling axis. (A) Heatmap of Spearman coefficient correlating protein expression levels of various oncogenic signaling proteins and NHE9 transcript in the TCGA pan-cancer dataset. (B) Correlative analysis between p-STAT3 (Y705) protein level and NHE9 transcript in the TCGA glioma patient cohort. (Correlation coefficient: 0.19; ****P-value < 0.0001) (C) Schematic of the IGF-1R-JAK2-STAT3 signaling axis. (D) Representative western blot of p-STAT3 and STAT3, and densitometry quantification (E). (F) Representative western blot GBM 612 scramble or NHE9KD probing for p-STAT3 and STAT3 and averages of densitometry quantification of 3 independent transfections are shown (G). (H)–(J) Western blotting analysis with time course for the indicated time points in minutes, and 25 h postligand treatment with insulin in GBM 276 GFP or NHE9-GFP as described in Methods. Blots were probed for (H) pro-IGF-1R, IGF-1R, p-IR/IGF-1R, p-IGF-1R (Y1216), p-IGF-1R (Y1135), (I) p-JAK2, JAK2, p-STAT3, STAT3, (J) Nanog (D73G4), Nanog (1E6C4), OCT4A, and GAPDH. (K) qPCR analysis for Oct4 and Nanog transcript in GBM 276 GFP or NHE9-GFP with or without STAT3 inhibitors, Cryptotanshinone (Crypto) or Stattic.

To identify potential links between NHE9 and oncogenic pathways functioning downstream of RTK activation, we screened the TCGA pan-cancer studies dataset for signaling protein expression against NHE9 transcript levels. STAT3 is widely recognized as a master regulator and driver of transforming events leading to GBM, and has been studied as a prognostic marker and therapeutic target in GBM (28, 30, 31) and other cancers (27, 32). We noted that p-STAT3 (Y705) had the highest correlation with NHE9 expression (Fig. 3A). The same analysis was performed in glioma patients for pSTAT3 (Y705), again revealing significant correlation with NHE9 (Fig. 3B). These observations identified STAT3 signaling as a potential downstream mediator of NHE9 in GBM (Fig. 3C).

We tested this hypothesis by directly probing STAT3 phosphorylation in GBM 276 and 612 cells in response to NHE9 overexpression and KD, respectively. Western blotting of cell lysates revealed that ectopic expression of NHE9 significantly elevated p-STAT3 (pY705; Fig. 3D and E), whereas attenuation of NHE9 expression led to corresponding decrease in STAT3 phosphorylation (Fig. 3F and G). These findings suggest a causal relationship between NHE9 expression levels and STAT3 signaling that warranted further analysis.

We used western blotting to query the IGF-1R signaling cascade (Fig. 3C) in response to insulin (25 μg/ml) treatment of GBM 276 cells. Total IGF-1R expression was elevated in transgene expressing GBM 276 NHE9+ cells, compared to GFP-transfected control (276 GFP) at all time points after ligand treatment, with significant turnover of the receptor observed at 25 h (Fig. 3H). The activated form of IGF-1R was observed by using 3 different antibodies that detect p-IR/p-IGF-1R (Y1150/1151 and Y1135/1136, respectively), p-IGF-1R (Y1316), and p-IGF-1R (Y1135), respectively. In each of the antibody blots, p-IGF-1R level in response to insulin addition was enhanced in NHE9+ cells but returned to nonstimulated levels by 25 h (Fig. 3H). Similarly, time-dependent increase of p-STAT3 and p-JAK2 was observed following insulin addition, with higher phosphorylation levels in GBM 276 NHE9+ cells (Fig. 3I). Remarkably, persistent phosphorylation at 25 h postinsulin addition was suggestive of prolonged downstream effects of RTK activation. Total levels of STAT3 and JAK2 remained unchanged in both cell lines, again pointing to a post-translational effect. Later in the time course, expression of 2 downstream targets of the JAK-STAT pathway, Oct4 and Nanog, was achieved in response to STAT3 elevation, including and up to 25 h after insulin treatment (Fig. 3J). Target gene expression was significantly enhanced in NHE9+ cells, whereas the housekeeping protein GAPDH was similar under all conditions (Fig. 3J).

We used pharmacological blockers to validate the role of STAT3 signaling in the transcriptional output of Oct4 and Nanog downstream from NHE9. Inhibition of p-STAT3 with Stattic and Crypto (at 6.25 µM and 10 µM, respectively) successfully abrogated the effect of NHE9 on Oct4 and Nanog expression as evidenced by their mRNA (Fig. 3K) and protein levels (Figure S2A, Supplementary Material). The efficacy of these inhibitors and their dose-dependent ability to decrease p-STAT3 levels in GBM 276 was confirmed (Figure S2B–D, Supplementary Material).

### NHE9 induces pluripotency genes and suppresses differentiation markers

**Fig. 4.**
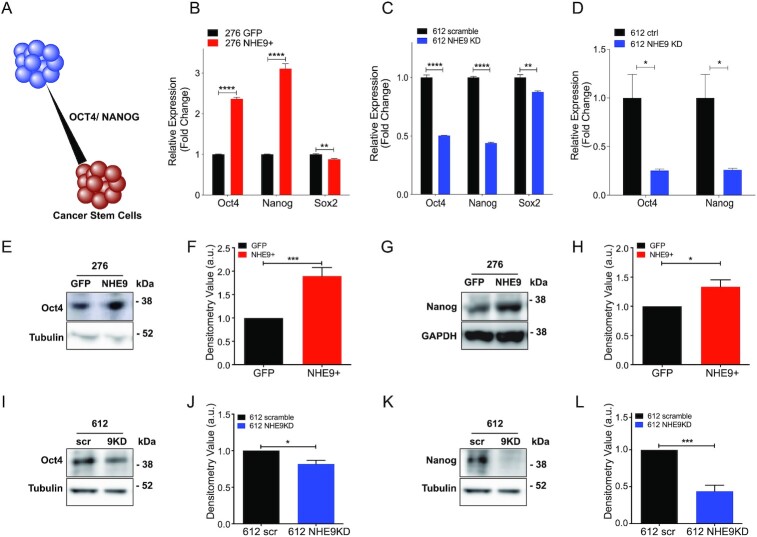
NHE9 increases stemness gene expression. (A) Schematic showing that Oct4 and Nanog shift distribution of cell state toward cancer stem cells (red), relative to differentiated cells (blue). (B)–(D) qPCR analysis of Oct4, Nanog, and Sox2 transcripts, as indicated. (B) GBM 276 GFP and NHE9-GFP (C) GBM 612 scramble and shNHE9 (NHE9 KD) (D) GBM 612 control siRNA or siNHE9-coated PBAE nanoparticles (NHE9 KD). Representative western blots for (E) Oct4 and (G) Nanog in GBM 276 GFP or NHE9-GFP and (F) and (H) averages of densitometry quantifications are shown. Figure 4(E) shows different sections of the same gel as Fig. 3(D) and has the same loading control, tubulin. (I)–(L) Representative western blots for (I) Oct4 and (L) Nanog in GBM 612 scramble or NHE9 KD and densitometry quantifications (J) and (L) are shown. Figure 4(I) and (K) are also different sections of the same gel and have the same tubulin loading controls. In all cases, averages of 3 independently transfected sets of cells are shown.

Oct4 and Nanog, also known as pluripotency factors, are transcription factors highly expressed in embryonic stem cells (33), and capable of reprogramming cells of committed lineage back to their primitive, stem cell state (Fig. 4A). These reprogramming factors are also highly expressed in malignancies such as GBM, where they confer worse prognosis in patients by eliciting therapeutic resistance and disease recurrence (34, 35). Consistent with the results from Fig. 3, we observed significant elevation of both Oct4 and Nanog transcripts in GBM 276 NHE9+ cells (Fig. 4B) and conversely, significant downregulation in GBM 612 NHE9 KD cells generated independently with lentiviral mediated shRNA (Fig. 4C) or siNHE9-loaded poly-β-amino ester (PBAE) nanoparticles (Fig.   4D). The siRNA delivery PBAE nanoparticles were specifically formulated to optimize siRNA delivery via reduction-sensitive bonds in the polymer backbone (Figure S3A and B, Supplementary Material), such that siRNA is released upon entering the cytosol. These nanoparticles have recently demonstrated safe and efficient siRNA delivery and gene KD in human GBM in vitro and in vivo and have promise as a translational therapeutic for nonviral NHE9 KD (36). The effect on Oct4 and Nanog was selective as expression of Sox2, which is also linked to stemness, showed modest, or no significant change (Fig. 4B and C). Western blot analysis was used to confirm that protein levels of Oct4 and Nanog were elevated in response to transgene NHE9 expression in GBM 276 (Fig. 4E–H), and reciprocally, decreased upon NHE9 KD in GBM 612 (Fig. 4I–L).

**Fig. 5.**
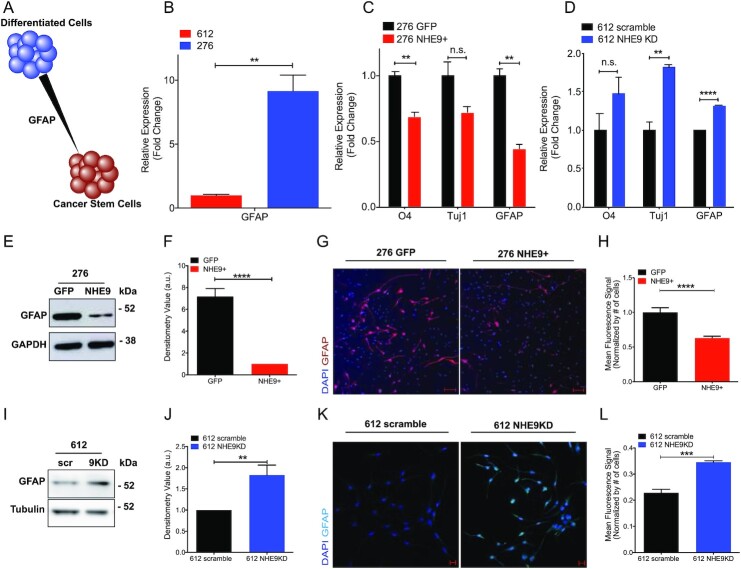
NHE9 decreases expression of differentiation markers. (A) Schematic showing that lineage markers such as GFAP shift cancer stem cells toward a differentiated state. (B)–(D) qPCR analysis for the indicated lineage markers, averaged from 3 biological replicates. (B) GBM 612 and GBM 275 (C) GBM 276 GFP or NHE9-GFP (D) GBM 612 scramble or NHE9 KD. (E) Representative western blot for GFAP in GBM 276 GFP or NHE9-GFP and (F) averages of densitometry quantification from 3 independent transfections are shown. (G) and (H) Representative immunofluorescence image for (G) GFAP (red) and DAPI (blue) in GBM 276 GFP and NHE9-GFP and (H) fluorescence signal quantification. (I) Representative western blot for GFAP in GBM 612 scramble or NHE9-GFP and (J) densitometry quantification. (K) and (L) Representative immunofluorescence image for (K) GFAP (light blue) and DAPI (blue) in GBM 612 scramble or NHE9 KD and (L) mean fluorescence signal quantification.

Next, we asked whether NHE9 expression was linked to reciprocal changes in differentiation markers that identify astrocyte lineage in GBM cells (Fig. 5A). We noted that transcript levels of the astrocytic marker glial fibrillary acidic protein (GFAP) were significantly higher in GBM 276 compared to GBM 612, corresponding to low and high endogenous NHE9, respectively (Fig. 5B). Further evaluation of matched NHE9 +/− pairs showed that expression of lineage markers for oligodendrocytes (O4), neurons (Tuj1), and astrocytes (GFAP) was decreased in GBM 276 NHE9+ cells (Fig. 5C), whereas NHE9 KD in GBM 612 increased transcript of all 3 lineage markers (Fig. 5D). Protein analysis recapitulated the qPCR results: decreased GFAP protein levels in GBM 276 NHE9+ cells as seen on western blots (Fig. 5E and F) and by confocal immunofluorescence microscopy (Fig. 5G and H). Conversely, NHE9 KD in GBM 612 increased GFAP level observed by western blotting (Fig. 5I and J) and immunofluorescence (Fig. 5K and L). Together, these results consistently demonstrate that NHE9 elicits stemness markers and may shift GBM to a cellular state closer to progenitor stem cells.

### Endosomal alkalinization is required for GBM stemness

**Fig. 6.**
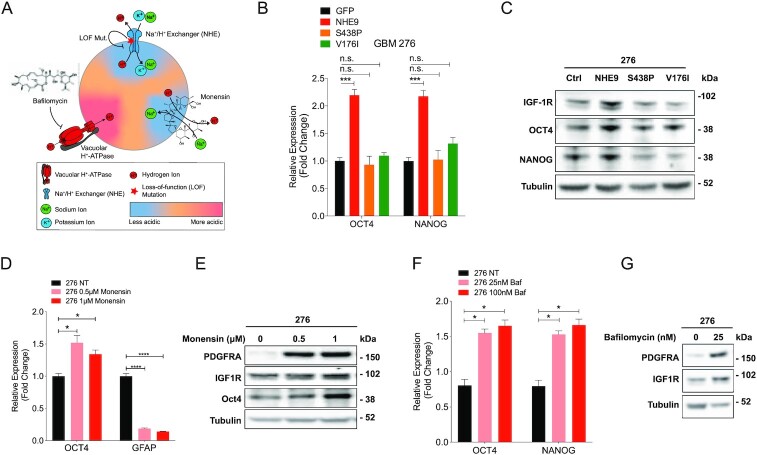
Endosomal pH regulates stemness in GBM cells. (A) Schematic of the 3 approaches used to query the role of endosomal pH. Loss of function (LOF) mutations in the Na^+^/H^+^ exchanger prevent endosomal alkalinization. Acidification by Vacuolar H^+^-ATPase is blocked by bafilomycin. The ionophore monensin mimics Na^+^/H^+^ exchange activity of NHE9. (B) qPCR analysis of Oct4 and Nanog transcripts in GBM 276 expressing vector (GFP), or the following GFP-tagged constructs: NHE9, S438P, or V176I. (C) Western blot of GBM 276 lysates expressing GFP (Ctrl) or the NHE9 constructs indicated, for IGF-1R, Oct4, and Nanog. (D) qPCR for Oct4 and Nanog transcripts in GBM 276 with no-treatment, 0.5 µM monensin, or 1 µM monensin for 16 h. (E) Western blot of PDGFRα, IGF-1R, and Oct4 in GBM 276 treated with monensin at the concentrations shown for 16 h. (F) qPCR analysis for Oct4 and Nanog transcripts in GBM 276 treated with bafilomycin at the concentrations shown for 6 h. (G) Western blot for PDGFRα and IGF-1R in GBM 276 treated with bafilomycin as shown for 6 h.

We used both genetic and chemical approaches to manipulate endosomal pH (Fig. 6A) and evaluate stemness phenotypes. First, we considered whether ion transport by NHE9 was required for driving stemness characteristics. In addition to the membrane embedded transporter domain, Na^+^/H^+^ exchangers have a cytosolic tail (∼150 aa), known to bind regulatory factors and scaffold signaling proteins that are implicated in tumorigenesis (21, 37, 38). Thus, the ability of NHE9 to drive stemness may be due to ion transport activity and/or signaling through the cytosolic tail. To begin to distinguish between these roles, we deployed previously characterized, autism-associated missense NHE9 mutants (S438P and V176I) that have normal protein expression and endosomal localization but have lost ion transport activity and thus fail to alkalinize the endosomal lumen (Fig. 6A) (24). We validated localization of wild-type and mutant NHE9 to the recycling endosome by co-localization with transferrin in GBM 276 (Figure S4A, Supplementary Material). Only wild-type NHE9, but not S438P and V176I mutants was able to increase Oct4 and Nanog transcript (Fig. 6B). This finding was confirmed by western blotting: mutants S438P and V176I failed to phenocopy wild-type NHE9 in stabilizing IGF-1R and failed to elevate Oct4 and Nanog (Fig. 6C). These observations point to the importance of endosomal Na^+^/H^+^ exchange activity in tumor initiation.

The pH of the recycling endosome is precisely tuned by a balance between inward proton pumping through V-ATPase and outward proton leak by NHE9 (Fig. 6A). Thus, high levels of NHE9 expression seen in GBM cells corresponds to alkaline endosomes (20). To directly query the role of pH in GBM stemness, we used pharmacological approaches to alkalinize the endosomal lumen and bypass the requirement for NHE9, with the caveat that these agents are not compartment-specific. The ionophore monensin is a Na^+^/H^+^ exchanger mimetic (39, 40) (Fig. 6A). Acute application of monensin to GBM 276 cells caused a dose-dependent elevation of endosomal pH as expected (Figure S4B and C, Supplementary Material). Here, we show that monensin increased Oct4 transcript relative to vehicle control and caused a reciprocal decrease in GFAP levels (Fig. 6D). Furthermore, monensin also stabilized IGF-1R and PDGFRα, and increased Oct4 protein as seen by western blotting (Fig. 6E). Bafilomycin inhibits the H^+^ pumping V-ATPase to prevent acidification of endolysosomal compartments (Fig. 6A). We show that bafilomycin treatment of GBM 276 increased Oct4 and Nanog transcript levels (Fig. 6F) and stabilized PDGFRα and IGF-1R (Fig. 6G). Taken together, these findings reveal that alkalinization of endosomal pH is the underlying mechanism for NHE9-mediated stemness in GBM.

### NHE9 induces self-renewal capacity in vitro and tumor formation in vivo

**Fig. 7.**
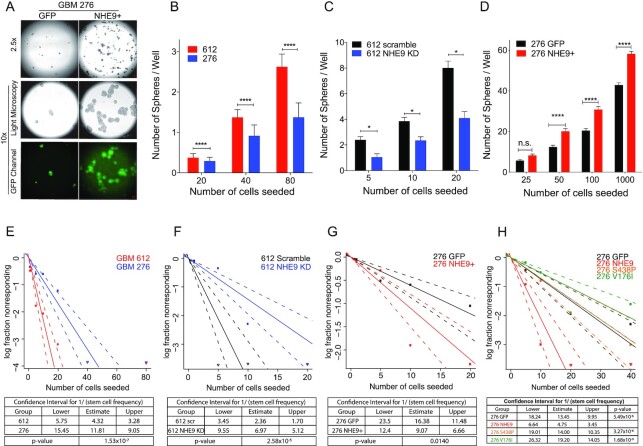
NHE9 increases self-renewal in GBM cells. (A) Light microscopy and fluorescence images of tumorspheres formed from GBM 276 GFP or NHE9-GFP cells in suspension in serum-free media on a noncoated petri dish after 2 weeks of culturing (scale bars, 50 µm). (B)–(D) Number of tumorspheres per well in a 96-well plate at various seeding dilutions as indicated in cell numbers for (B) GBM 612 or GBM 276 (C) GBM 612 scramble or NHE9 KD (D) GBM 276 GFP or NHE9-GFP. (E)–(H) ELDA for estimated stem cell frequency and CI for (E) GBM 612 or GBM 276, (F) GBM 612 scramble or NHE9KD, (G) GBM 276 GFP or NHE9-GFP, and (H) GBM 276 GFP, NHE9-GFP, NHE9-GFP (S438P), or NHE9-GFP (V176I).

The functional consequence of increased stemness is typically quantified in vitro using tumorsphere formation as a means to assess self-renewal capacity of cells. As seen in the fluorescence images, tumorspheres formed from GBM 276 cells transfected with NHE9-GFP were larger, consistent with our previous report that NHE9 increases tumor cell proliferation (20), and also more numerous than GFP vector-transfected control (Fig. 7A). Furthermore, the number of spheres per uncoated well correlated with seed density and NHE9 expression: for the same seed density, significantly more spheres were obtained from NHE9-high GBM 612 compared to NHE9-low GBM 276 (Fig. 7B). Tumorsphere formation capacity decreased after NHE9 KD in GBM 612 and increased after transgene NHE9 expression in GBM 276 (Fig. 7C and D). A quantitative estimate of stem cell frequency was determined by extreme limiting dilution assay (ELDA) (41). More progenitor cells capable of seeding tumorspheres were detected in GBM 612, relative to GBM 276 (Fig. 7E). Progenitor cell frequency decreased with NHE9 KD (Fig. 7F) and increased with transgene NHE9 expression (Fig. 7G). Furthermore, unlike wild-type NHE9, S438P and V176I mutants do not increase self-renewal capacity (Fig. 7H), and confirm the findings from Fig. 6 on the importance of Na^+^/H^+^ exchange activity in conferring stemness phenotypes.

**Fig. 8.**
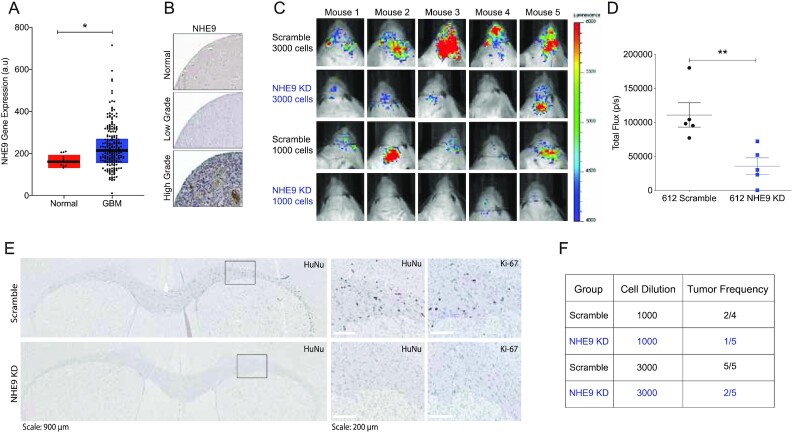
NHE9 increases tumor initiation in vivo. (A) Comparison of NHE9 (*SLC9A9*) mRNA expression between GBM tumor and normal samples from cBioPortal database (*P-value: 0.0423; Student's t test). (B) Representative immunohistochemistry staining for NHE9 protein from normal, LGG, and HGG, obtained from the Human Protein Atlas. (C) Bioluminescence signal from mice with orthotopic brain xenograft of luciferase-expressing 612 GBM tumor cells with or without NHE9-shRNA seeded with 3,000 or 1,000 cells. (D) Quantification of total bioluminescence flux from mice with 3,000 cells (**P-value: 0.0084; Student's t test). (E) Representative immunostaining images for Human Nuclei (HuNu) and Ki-67 from brain tissues injected with control or NHE9 KD cells with 3,000 cells. (F) Tumor frequency in mice injected with GBM 612 control or NHE9 KD cells with 1,000 and 3,000 cells.

Previously, we reported that NHE9 expression is elevated in most GBM subtypes, with highest levels in mesenchymal tumors (20). A small sample of GBM tumor stem cells (*n* = 22) relative to normal neural stem cells (*n* = 3) showed a 5-fold elevation of NHE9 transcript (20). Expanded analysis using cBioPortal shows elevated NHE9 expression in GBM, in comparison to nonmalignant brain samples (Fig. 8A). Statistical analysis (ANOVA) of NHE9 expression in 24 immunohistochemistry slides revealed significant variability between normal, low-grade glioma (LGG), and high-grade glioma (HGG) samples (*P* = 0.0185) as shown by the representative images in Fig. 8(B). In addition, student t test analysis of NHE9 staining between LGG and HGG patients showed significant difference (*P* = 0.0272).

To determine the contribution of NHE9 to tumor initiation in vivo, we performed orthotopic transplant of luciferase-expressing GBM 612 transfected with scramble RNA (control) or shNHE9 (KD) at low seed counts (1,000 and 3,000 cells). Figure 8(C) shows bioluminescence (BLI) images of mouse brains 10 weeks postxenograft. Quantitative luminescence analysis revealed significantly decreased light flux in mouse brains injected with NHE9 KD cells compared to control pointing to fewer tumor cells (*P* = 0.0084; 3,000 cells; Fig. 8D). Subsequently, brains were sectioned and stained for human nuclei (HuNu) and the cell proliferation marker Ki67 to evaluate tumor formation. Representative immunohistochemistry images are shown in Fig. 8(E). For control mice, immune-positive staining was found for both HuNu and Ki67 markers across the corpus callosum and dispersed throughout the brain parenchyma. Conversely, mice injected with NHE9 KD cells showed little or no staining. Total body weight of all mice was similar and remained mostly unchanged over 11 weeks (Figure S5A, Supplementary Material). After investigating all stained brain sections (Figure S5B and C, Supplementary Material) for viable, dividing human GBM cells, we confirmed decreased tumor frequency with NHE9 KD (Fig. 8F). Quantitative analysis of these data revealed statistically significant difference in tumor formation (*P* = 0.0185). These findings demonstrate that NHE9 not only elevates expression of genes associated with stemness and pluripotency, but also self-renewal capacity at a functional level, measured by tumorsphere formation in vitro and tumor initiation in vivo.

## Discussion

The recycling endosome is a critical hub for oncogenic receptor trafficking. There is accumulating evidence that dysregulation of endosomal trafficking in cancer contributes to the progression of malignancy. For example, quantitative proteomics of primary brain tumor samples revealed that the endocytic machinery is commonly down regulated in brain malignancies regardless of tumor grade and type (42). Intriguingly, loss of the RNA-binding protein QKI was shown to attenuate degradation of oncogenic growth factor receptors by down regulating biogenesis of endolysosomal compartments, leading to maintenance of self-renewal in glioma stem cells even in a growth factor-deficient environment (43). Here, we show that gain of NHE9 function and hypoacidification of the recycling endosome lumen increases recycling of oncogenic growth factor receptors and promotes their escape from degradation, resulting in tumor stemness phenotypes. In another study, a suboptimal environment of iron-deficiency in tumors was found to increase NHE9-mediated transferrin receptor surface recycling in a model of the blood–brain barrier (44). Thus, brain tumor cells adopt multiple mechanisms of altered endosomal trafficking to sustain oncogenic receptor density at the cell surface and scavenge scarce nutrients from the tumor microenvironment. Despite their recognized role in cell-environment signaling, recycling endosomes have not been therapeutically exploited, due to a lack of a specific and selective target.

We describe a mechanism of endosomal pH dysregulation through an imbalance of proton pump and leak pathways that allows multiple receptors to evade lysosomal degradation, expanding on our previous observation linking NHE9 to EGFR expression levels (20). By alkalinizing the endosomal lumen in GBM cells, NHE9 functionally recapitulates the role of E5 oncoprotein of human papilloma virus (HPV), the main cause of cervical cancer worldwide. E5 interacts with V-ATPase to inhibit proton pump activity, alkalinize endosomes and drive tumorigenesis (45, 46). However, tumorigenic alterations in pH appear to be compartment-specific: in a model of breast cancer, decreased expression of NHE6 correlated with hyperacidification of early endosomes resulting in drug sequestration and chemotherapeutic resistance (21). Consistent with the oncogenic role of acidic pH in early endosomes, increased V-ATPase activity in this compartment leads to increased shedding of oncogenic extracellular vesicles in GBM cells (47). In a pancreatic cancer cell model, elevated expression of the *trans*-Golgi network isoform NHE7 resulted in hyperacidification of Golgi lumen and activation of plasma membrane Na^+^/H^+^ exchange (22), mimicking NHE1-mediated regulation of cytosolic pH and extracellular acidification common to a wide range of cancer types.

Although hyperactivation of oncogenic RTK signaling in GBM tumors has been extensively appreciated, efforts in therapeutic targeting of single receptors such as EGFR and PDGFR have largely failed in clinical trials due to receptor-heterogeneity and redundancy. Finding a drug target that inhibits multiple oncogenic receptors may circumvent this problem. Thus, a druggable target such as NHE9 that controls both stemness and pan-receptor clearance in GBM cells could circumvent shortcomings in current therapy. Our findings could spur a search for inhibitors that selectively target the NHE9 isoform.

The link between pH and pluripotency is intriguing but not fully explored. Few studies suggest that exposure to extracellular acidity can induce stemness in tumors (14, 48), however, the role of organellar pH is unclear. Furthermore, the link between extracellular and endosomal pH, if any, should be investigated in future studies. This work represents a first step in evaluating the role of NHE9 in mediating stemness, and as such, has some limitations. Strictly speaking, stem cells are capable of self-renewal as well as multilineage differentiation, therefore, a thorough evaluation of molecular and functional lineage and stem cell markers (13, 49) associated with NHE9 in GBM may be helpful. While significant effort has been invested in deciphering the role of extracellular pH in various malignancies, our findings revealing the previously unexplored role of endosomal pH in stemness warrant further study.

## Methods

### GBM cell culture

All GBM tumor samples and subsequent cell line derivations were performed with written consents from patients through Quiñones laboratory, observing Institutional Review Board guidelines as previously described. GBM stem cells with tumorsphere forming capabilities, GBM 276 and GBM 612, were cultured in DMEM/F12 (1:1), 1% HyClone Antibiotic Antimycotic Solution (Thermo Scientific), Gibco B-27 serum free Supplement (Thermo Scientific 17504044), 20 ng/ml EGF, and 20 ng/ml FGF. For ligand treatment experiments, Gibco B27 serum-free supplement, minus insulin (Thermo Scientific A1895601) was used. For adherent culture, dishes were coated with Laminin (Sigma-Aldrich L2020). Coating was done in DMEM/F12 basal media for at least 2 h or overnight, and for every 1 cm^2^ of surface area, 1 μl of laminin was added. Drugs used in this study are as follows: Cycloheximide (Cell signaling #2112), Bafilomycin A1 (InvivoGen CAS # 88899-55-2), MG-132 (InvivoGen CAS # 133407-82-6), Cryptotanshinone (Selleckchem Catalog No. S2285), Stattic (Selleckchem Catalog No. S7024), and S3I-201 (Selleckchem Catalog No. S1155).

### Plasmids and oligos

Full-length mNHE9 was cloned into FuGW lentiviral vector as previously described. HsNHE9 targeting short hairpin RNA (shRNA) (5’-CCGGCCCTCCAT TAAGGAGAGTTTTTCAAGAGAAAACTCTCCTTAATGGAGGTTTTTC-3’) and scramble control (Sigma-Aldrich) (5’- CAACAAGATGAAGAGCACCAA-3’) were cloned into pLKO.1 lentiviral vector with ampicillin and puromycin-resistance genes for bacteria selection and mammalian cell selection, respectively. Constitutively active STAT3 (EF.STAT3C.Ubc.GFP, plasmid #24983) was purchased from Addgene. siRNA oligos targeting human NHE9 were purchased from Origene Technologies (Rockville, MD) with the following sequences: SR317216A rCrCrArCrUrUrUrCrArArGrCrUrUrArGrUrArGrGrArCrCAT, SR317216B rCrCrCrUrCrUrCrUrArUrUrArUrGrCrArArArUrGrArArUTT, and SR317216C rGrCrArGrGrArUrArUrArGrUrCrUrArArArGrArArGrArGAC. A comparison of KD efficiency is shown in Figure S3B (Supplementary Material) and SR317216C was determined to be the most effective NHE9-targeting siRNA.

### Endosomal pH measurement

Endosomal pH measurement was measured using pH sensitive FITC (50 µg/ml), normalized for uptake with pH insensitive Alexa Fluor 633 (50 µg/ml; Invitrogen) tagged to transferrin (100 µg/ml) as described previously (20). Where indicated, treatment of GBM 276 cells with monensin (10 and 50 µM) was for 1 h. Adherent cells were washed with PBS once, and treated with tagged transferrin at 37°C for 55 min. Transferrin uptake was stopped by chilling the cells on ice. Excess transferrin was removed by washing with ice-cold PBS, whereas cell surface-bound transferrin was removed by an acid wash in PBS at pH 5.0 followed by a wash with PBS at pH 7.4. The fluorescence intensity for both dyes was measured for at least 5,000 cells by flow cytometry using the FACSAria (BD Biosciences) instrument and the average intensity of the cell population was recorded. A pH calibration curve was generated in buffers at different pH (4.5, 5.5, and 6.5) in the presence of K^+^/H^+^ ionophores, nigericin (10 μM) and valinomycin (10 μM) by following manufacturer's protocol from the Intracellular pH Calibration Buffer Kit (Invitrogen).

### ELDA

The cells were enzymatically dissociated with Accutase and were filtered into 5 ml polystyrene round-bottom tube with cell strainer cap (#352235, Falcon). In order to increase the accuracy of the number of cells seeded, the FACS Aria sorter at the Ross Flow Cytometry Core at the Johns Hopkins was utilized to seed indicated number of cells in each well of uncoated 96-well plate. The cells were cultured in sphere form for 2 weeks, and the number of spheres was determined for each well. Analyses of self-renewal ability and stem cell frequency were performed using the algorithm on the following website: http://bioinf.wehi.edu.au/software/elda/index.html.

### Intracranial implantation of GBM cells

In vivo methods were carried out in accordance to the JHU Institutional Handbook on the Use of Experimental Animals. Intracranial implantation of GBM612 cells (transduced with control and NHEKD vectors) was carried out using 2 different dilutions of 1,000 cells (*n* = 9) and 3,000 cells (*n* = 10). Orthotopic implantation of cells was done in 6-week-old male athymic nude mice. Animals were anesthetized with ketamine HCl (80 mg/kg) and xylazine HCl (8 mg/kg) via IP injection. Mice were then positioned in a stereotactic frame (KOPF model 2006 small animal stereotaxic instrument with digital display console) with a surgical microscope (Zeiss surgical microscope MPMI-1 FC). A linear midline incision of the scalp was made to expose the calvarium. Different dilutions of GBM612 cells were stereotactically injected into the striatum *(X: 1.5 mm, Y: 1.34 mm*,and*Z: -3.5 mm from Bregma)*. After cell injection, the needle was carefully retracted, and the skin was closed with surgical glue. Mice were placed on a heating pad to speed recovery. Tumor formation and growth was confirmed by weekly BLI. After 11 weeks of surgical implantation of tumors, mice were intracardially perfused with 4% paraformaldehyde. Tumor formation was determined by immunohistochemistry.

### Immunohistochemistry

Mouse brain tissue was fixed in 4% PFA and embedded in paraffin. Formalin-fixed paraffin embedded sections (10 µm thick) were deparaffinized and rehydrated, followed by antigen recovery. A total of 3% hydrogen peroxide was used to block endogenous peroxidase activity, followed by Rodent Block M (Biocare) after which slides were washed in phosphate-buffered saline and stained against HuNu (MAB4190, 1:250, Millipore) and Human specific Ki67 (MAB4383, 1:500, Millipore), followed by secondary antibody Envision+ antimouse labeled-polymer (Dako/Agilent). Slides were counterstained using Gill's I hematoxylin (Richard-Allen). Stained slides were imaged using Aperio AT2 scanner and analyzed by Aperio ImageScope software.

### Statistical analysis

Experiments were conducted in triplicates of independently generated transfections or cultures to meet the standard for statistical power. For quantitative PCR analysis, ΔΔCt values in triplicate were calculated by Applied Biosystems 7500 analysis program. Those values were normalized in comparison to the control, which then was analyzed by using 2 tailed Student's t test. For western blotting analysis and flow cytometry, normalized densitometry and mean fluorescent values, respectively, were analyzed with 2 tailed Student's t test. For all quantitative data graphed and presented in the current research, mean values ± SDE were plotted. Statistical significance was assumed when the *P*-value was lower than 0.05. (nonsignificant: *P*-value > 0.5, *: *P*-value < 0.05, **: *P*-value < 0.01, **: *P*-value < 0.001, and ****: *P*-value < 0.0001).

## Supplementary Material

pgac013_Supplemental_Files
