# Correction to: The endosomal pH regulator NHE9 is a driver of stemness in glioblastoma

**DOI:** 10.1093/pnasnexus/pgad456

**Published:** 2024-01-30

This is a correction to: Myungjun Ko, Monish R Makena, Paula Schiapparelli, Paola Suarez-Meade, Allatah X Mekile, Bachchu Lal, Hernando Lopez-Bertoni, Kristen L Kozielski, Jordan J Green, John Laterra, Alfredo Quiñones-Hinojosa, Rajini Rao, The endosomal pH regulator NHE9 is a driver of stemness in glioblastoma, *PNAS Nexus*, Volume 1, Issue 1, March 2022, pgac013, https://doi.org/10.1093/pnasnexus/pgac013

During a retroactive audit conducted by *PNAS Nexus*, it was discovered that this paper was missing a statement acknowledging compliance with the *PNAS Nexus* Human and Animal Participants and Clinical Trials policy:

The study protocol involving human participants was approved by the Johns Hopkins Hospital and the Mayo Clinic Institutional Review Boards (IRB). Glioma cell lines and tissues were derived from patient samples that were obtained and processed at the Johns Hopkins Hospital and the Mayo Clinic under the approval of the IRB. All animal protocols were approved by the Mayo Clinic Animal Care Institutional and Use Committee.

This error has been corrected in the original article.

