# Supplementary material for: The endosomal pH regulator NHE9 is a driver of stemness in glioblastoma
Source: PNAS Nexus. 2022 Mar 9;1(1):pgac013. doi: 10.1093/pnasnexus/pgac013 (PMC8974362; doi:10.1093/pnasnexus/pgac013)
Supplement: pgac013_Supplemental_Files [file pgac013_supplemental_files.zip › PNASNEXUS-PNASNEXUS-2021-00240-T-s01.pdf]

Supplemental Information for The Endosomal pH Regulator NHE9 is a Driver of Stemness in Glioblastoma, Ko et al.

Supplemental Methods

Nanoparticle synthesis and *in vitro* siRNA delivery: Bioreducible siRNA delivery PBAE polymer, termed R646, was synthesized as previously described (Fig. S3A) (52). Briefly, monomer bis(2-hydroxyethyl) disulfide (BR6) was synthesized via acrylation of bis(2-hydroxyethyl) disulfide using acryloyl chloride under anhydrous conditions (71). Polymerization of monomer BR6 with side chain monomer 4-amino-1-butanol (S4), followed by polymer end-capping with 2-((3-aminopropyl)amino)ethanol (E6), yielded polymer BR6-S4-E6 (R646). Unreacted monomers were removed via precipitation of the polymer with diethyl ether, and R646 was dissolved in anhydrous DMSO at 100 mg/mL, and stored at -20°C with desiccant. In order to form nanoparticles for knockdown experiments, R646 and siRNA were separately diluted in 25 mM sodium acetate (pH=5) and mixed in a 1:1 v/v ratio. The polymer and siRNA were allowed to self-assemble into nanoparticles for 10 min before they were added to the cell culture media at concentrations of 270 μg/mL and 120 nM, respectively. Nanoparticles remained in the cell culture media for 2 hours at 37°C, after which the media containing nanoparticles was removed and replaced with fresh media. The polymer structure was validated by H^1-NMR and the molecular weight (M_N = 3151 g/mol; M_W = 4004 g/mol) and polydispersity (1.271) was determined by gel permeation chromatography as previously reported (72). The nanoparticle hydrodynamic size as characterized by nanoparticle tracking analysis was 137 ± 6 nm, the zeta potential as characterized via electrophoretic mobility was 18 ± 1 mV, and the nanoparticle morphology was spherical as characterized via transmission electron microscopy (TEM) as previously reported (52).

Production of lentivirus and cell transduction: pCMV-dR8.9 (8.8μg), pMD2.G (2μg) lentivirus packaging plasmids, and NHE9 plasmids (10 μg) were co-transfected with pLKO.1-shNHE9 in HEK293FT cells with lipofectamine 3000 (ThermoFisher Scientific) according to manufacturer's protocol. Media was changed the next day, and virus was collected 48h, 72h, and 96h after transfection. The collected media containing virus was filtered through 0.45μm pore filter and mixed with Lenti-X concentrator (Clontech) as indicated by manufacturer's instruction. Centrifugation was preformed overnight incubation at 4°C at 3300 rpm for 15 mins. The pellet was resuspended in PBS and was either used immediately or frozen at -80°C for later use. For glioblastoma cell transduction, cells were seeded on laminin-coated dishes and infected with lentivirus. In case of shRNA constructs, cells were incubated with lentivirus for 48 h in low volume of media. Subsequently, GBM612 cells were recovered in GBM complete media for 6 h and were selected with puromycin (1μg/ml). GBM 276 cells transduced with FuGW and mNHE9-GFP virus were sorted by fluorescence-activated cell sorting (FACS) for GFP positive cells to be used for subsequent assays and analysis.

Quantitative PCR analysis: RNA was extracted from cultured cells using the RNeasy Mini kit (#74104, Qiagen) according to the manufacturer's instructions. Complementary DNA was synthesized using the high-Capacity RNA-to-cDNA kit (#4387406, Applied Biosystems). Quantitative PCR analysis was performed using the 7500 Real-Time PCR system (Applied Biosystems) with Taqman 2x Fast universal PCR Master Mix (#4304437, Applied Biosystems). Taqman gene expression assay probes used in this study are: Human: NHE9 (SLC9A9), Hs00543518; NHE6 (SLC9A6), Hs00234723; Oct4 (POU5F1), Hs00999632; Nanog, Hs04399610; Sox2, Hs01053049; c-Myc, Hs00811069;KLF4, Hs00358836 ;Olig2, Hs00300164; Nestin (NES), Hs04187831; CD133(PROM1), Hs01009257; GFAP, Hs00909233;TUJ1

(TUBB3), Hs00801390;O4 (FOXO4), Hs00936217; IGF1R, Hs00609566; IR, Hs00961557; PDGFRA, Hs00998018; GAPDH, Hs02786624. The Ct (cycle threshold) values were determined first and subsequently normalized to GAPDH expression (endogenous control) to obtain ΔCt value for each sample. These ΔCt values were compared to the control ΔCt values and acquired ΔΔCt values. Fold changes were then calculated using the equation: expression fold change = $2^{-\Delta\Delta Ct}$. Each experiment was performed at least three times independently.

Immunoblotting: Cells were lysed using Pierce™ RIPA buffer (#89900, Thermo Scientific) supplemented with Protease/phosphatase Inhibitor cocktail (100x) (5872S, Cell Signaling Technology). Cells were sonicated for 15-20 seconds each sample and rotated for 15 min at 14,000 rpm at 4°C. Protein concentration was determined by the BCA assay. For each sample, 15-30 μg of protein was mixed with 10x reducing buffer and 4x loading buffer. The samples were heated for 10 min at 70 °C and separated on 10 or 12 well NuPage™ 4-12% Bis-Tris Gel (NP0322BOX or NP0321BOX) under reducing conditions, and then transferred onto activated PVDF membranes. Ponceau staining was utilized to confirm protein transfer. The membranes were blocked with either 5% milk or 5% BSA overnight at 4°C. The membranes were subsequently incubated with primary antibodies overnight at 4°C, followed by 1h incubation with HRP-conjugated secondary antibodies. SuperSignal™ West Pico PLUS Luminol/Enhancer solution and Stable Peroxide solution were mixed with 1:1 ratio and were introduced to the membranes for HRP activation. For imaging, digital Amersham Imager 600 system was used. Densitometric quantification and image processing was performed with ImageJ software or FIJI. Human Phospho-RTK Array Kit (ARY001B, R&D Systems) was used according to the manufacturer's instructions and the blots were imaged and quantified in the same way as western blots. Antibodies used for immunoblotting is as follows: NHE9 (*SLC9A9*) Rabbit PolyAb (Proteintech Group Cat.

13718-1-AP), Oct-4A (C30A3) Rabbit mAb (Cell signaling #2840), Nanog (D73G4) XP Rabbit mAb (Cell signaling #4903), GFAP (GA5) Mouse mAb (Cell signaling #3670), GFP (Cell signaling #2555), STAT3 (124H6) Mouse mAb (Cell signaling #9139), p-STAT3 (Tyr705) (D3A7) XP Rabbit mAb (Cell signaling #9145), p-JAK2 (Tyr1007/1008) (C80C3) Rabbit mAb, JAK2 *(D2E12*) XP Rabbit mAb, Insulin Receptor β (4B8) Rabbit mAb (Cell signaling #3025), p-Insulin Receptor β (Tyr1345) (14A4) Rabbit mAb (Cell signaling #3026**)**, *IGF-I Receptor* β (D23H3) Rabbit mAb (Cell signaling #9750), p-IGF-I Receptor β (Tyr1316) Antibody (Cell signaling #6113), p-IGF-I Receptor β (Tyr1135) (DA7A8) Rabbit mAb (Cell signaling #3918), p-IGF-I Receptor β (Tyr1135/1136)/Insulin Receptor β (Tyr1150/1151) (19H7) Rabbit mAb (Cell signaling #3024), PDGF Receptor *α (D1E1E)* XP Rabbit mAb (Cell signaling #3174), p-PDGF Receptor α (Tyr1018) Antibody (Cell signaling #4547)*,* and GAPDH (D4C6R) Mouse mAb (Cell signaling #97166).

Immunofluorescence: PHEM buffer was prepared (60mM PIPES, 25mM Hepes, 10mM EGTA, and 2mM $MgCl_2$ at pH 6.8). Cells were cultured on glass coverslips and were rinsed with PBS and pre-extracted with 1X PHEM buffer, 8% sucrose and 0.025% saponin. Cells were fixed with 4% paraformaldehyde in 1x PBS for 30 min and were washed with PBS 3 times for 5 min each. After blocking in 1% BSA in PBS with 0.025% saponin, cells were incubated with primary antibody in 1% BSA in 1x PBS with 0.025% saponin overnight at 4°C. Cells were rinsed with 0.2% BSA in 1x PBS 3 times for 10 min each and were then incubated with a fluorescent secondary antibody (1:1000) in 1% BSA in 1x PBS with 0.025% saponin for 30 min at RT. Coverslips were washed 3 times for 5 min each with 0.2% BSA in 1xPBS and mounted onto slides with ProLong(R) Gold Antifade with DAPI Molecular Probes (8961S, Cell Signaling Technology). Images were taken with Zeiss LSM780-FCS Single point, laser scanning confocal microscope and the obtained

images were analyzed using ImageJ. Antibodies used for immunofluorescence are as follows: Anti-Mouse IgG (H+L), F(ab')$_2$ Fragment (Alexa Fluor 647 Conjugate) (Cell signaling #4410), Anti-Rabbit IgG (H+L), F(ab')$_2$ Fragment (Alexa Fluor 555 Conjugate) (Cell signaling #4413), IGF-I Receptor β (D23H3) Rabbit mAb (Cell signaling #9750), and GFAP (GA5) Mouse mAb (Cell signaling #3670).

Supplemental Figures S1-S5

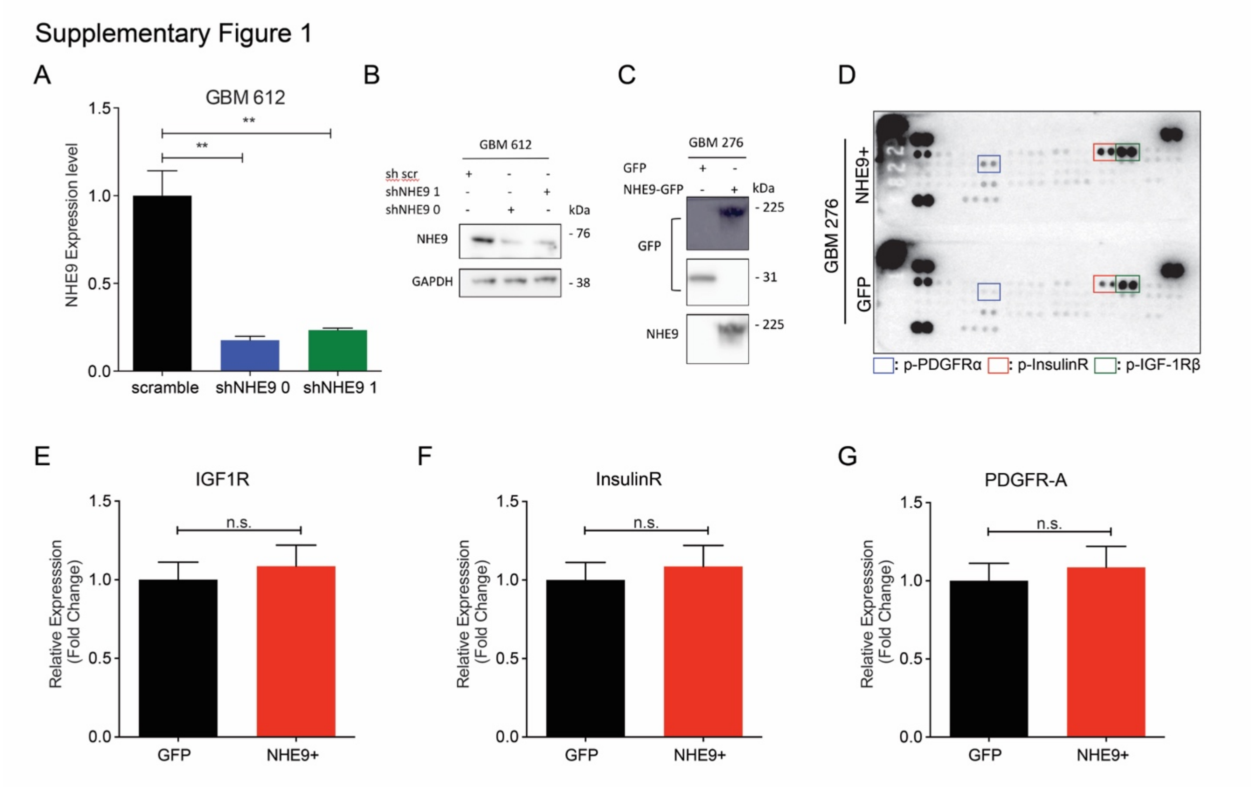

Figure S1: **Effect of NHE9 expression in GBM on receptor tyrosine kinases**

Relates to Figure 1.

(A) qPCR for NHE9 transcript in GBM 612 cells following treatment with two targeting shRNA constructs, relative to scramble control. ***p*-value: 0.0046 - Student's *t*-test; ***p*-value: 0.0057, Student's *t*-test). (B) Western blot of NHE9 of samples from A. (C) Western blot for GFP (top two panels) and NHE9 (bottom panel) in GBM 276 cells transfected with GFP or NHE9-GFP. (D) phosphorylated RTK dot blot overlaid with cell lysate from GBM 276 transfected with GFP or

NHE9-GFP. Boxed samples show p-RTKs excerpted in Fig. 1C, identified as shown by colored boxes, and chosen for study. (E-G) qPCR analysis for transcript levels of (E) IGF-1Rβ, (F) InsulinR, and (G) PDGFRα.

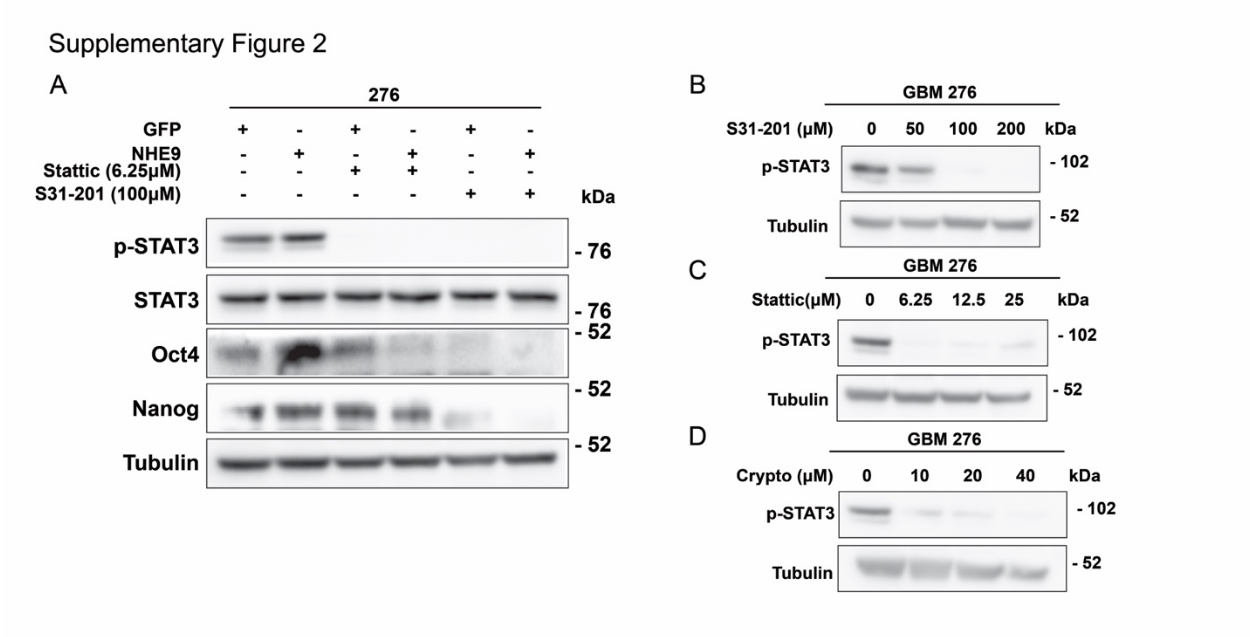

Figure S2: Analysis of STAT3 and JAK2 role in GBM

Relates to Figure 3.

(A) Western blot for p-STAT3, STAT3, Oct4, and Nanog proteins in GBM 276 GFP and NHE9-GFP, with STAT3 inhibitors, Stattic or S31-201 as indicated. (B-D) Western blot for p-STAT3 in GBM 276 with STAT3 inhibitors at increasing concentrations: (B) 0, 50, 100, and 200 µM of S31-201; (C) 0, 6.25, 12.5, and 25 µM of Stattic; and (D) 0, 10, 20, and 40 µM of Crytptanshinone.

Supplementary Figure 3

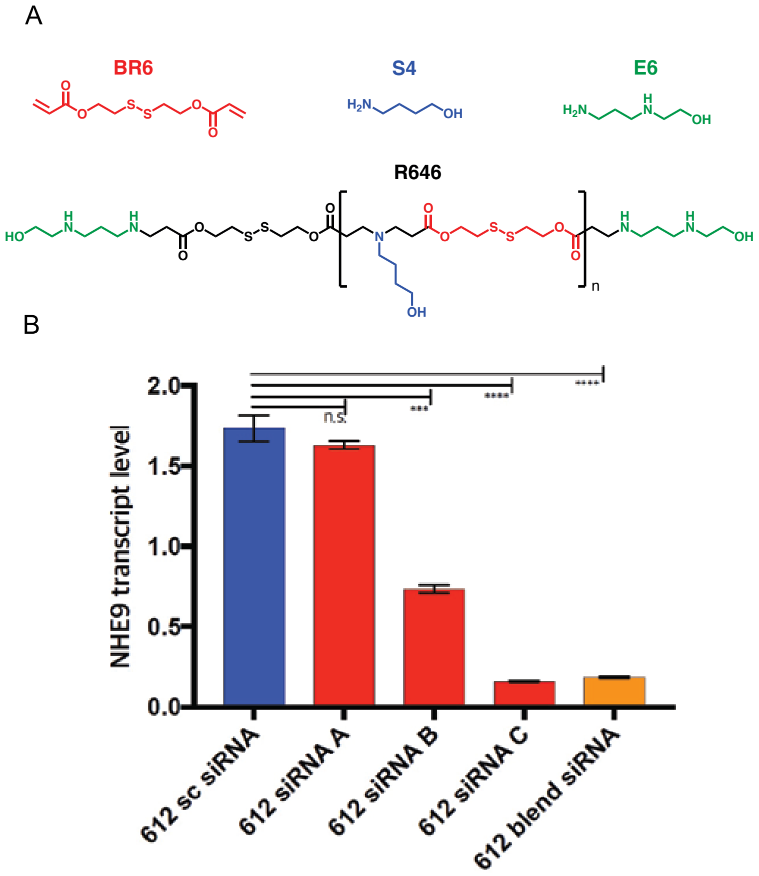

Figure S3: Chemical structure of siRNA delivery polymer.

Relates to Figure 4.

(A) The chemical structure of the poly-β-amino ester (PBAE) polymer used to generate NHE9 KD cells is shown. It is synthesized via polymerization of monomers BR6 and S4, followed by polymer endcapping with monomer E6. Disulfide bonds in the polymer backbone enable polymer degradation in the reducing environment of the cytosol, which optimizes siRNA delivery and targeted release. (B) PBAE-mediated knockdown efficiency of NHE9 in GBM 612 with three different siRNA oligos are compared, along with their blended combination (blend) and a scrambled control (sc).

Supplementary Figure 4

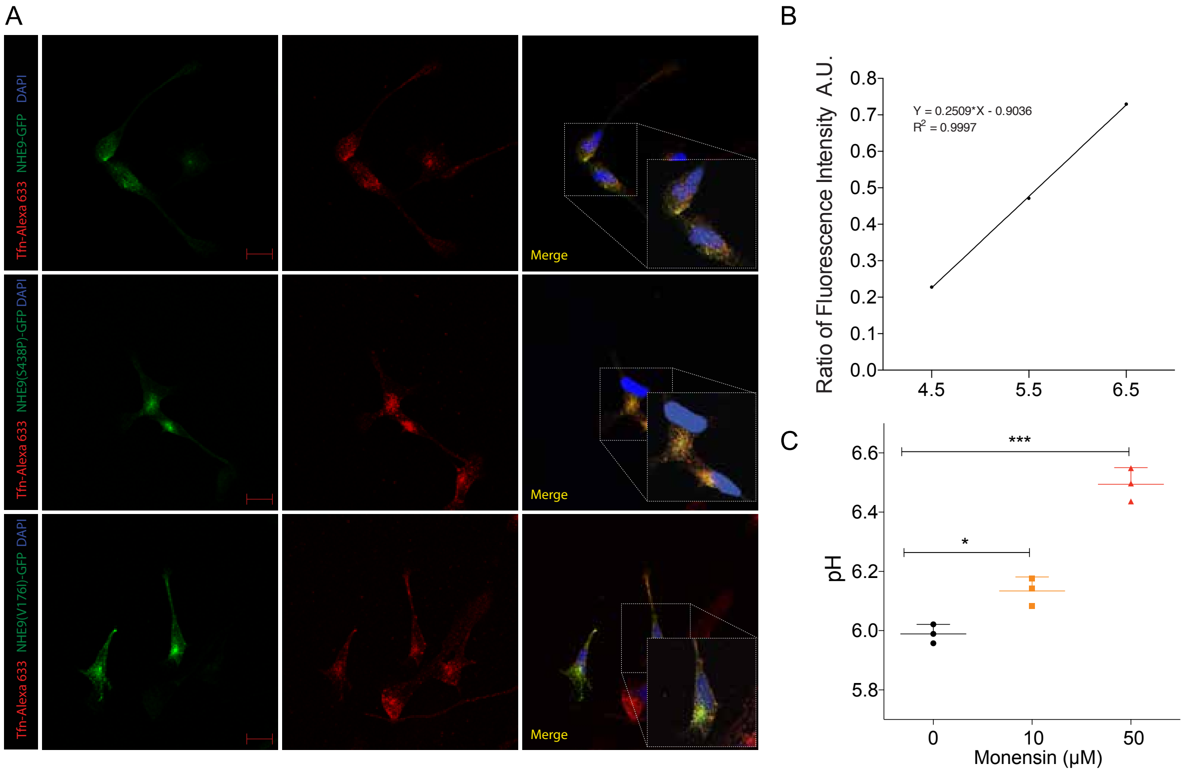

Figure S4: Role of endosomal pH in GBM

Relates to Figure 6.

(A) Immunofluorescence images of GBM 276 cells expressing NHE9-GFP, NHE9 (S438P)-GFP, or NHE9 (V176I)-GFP (Green) after 1hr treatment with Transferrin conjugated with Alexa 633 (Red). Nuclei are stained with DAPI (blue). Inset shows merged images; scale bars, 10 μm. (B) Calibration of fluorescence ratio intensity of transferrin tagged FITC and Alexa Fluor 633 in GBM 276 cells using buffers of indicated pH, as described in Methods. (C) Recycling endosome pH in GBM 276 cells treated with the indicated concentrations of monensin for 1 hour, as described in Methods.

Supplementary Figure 5

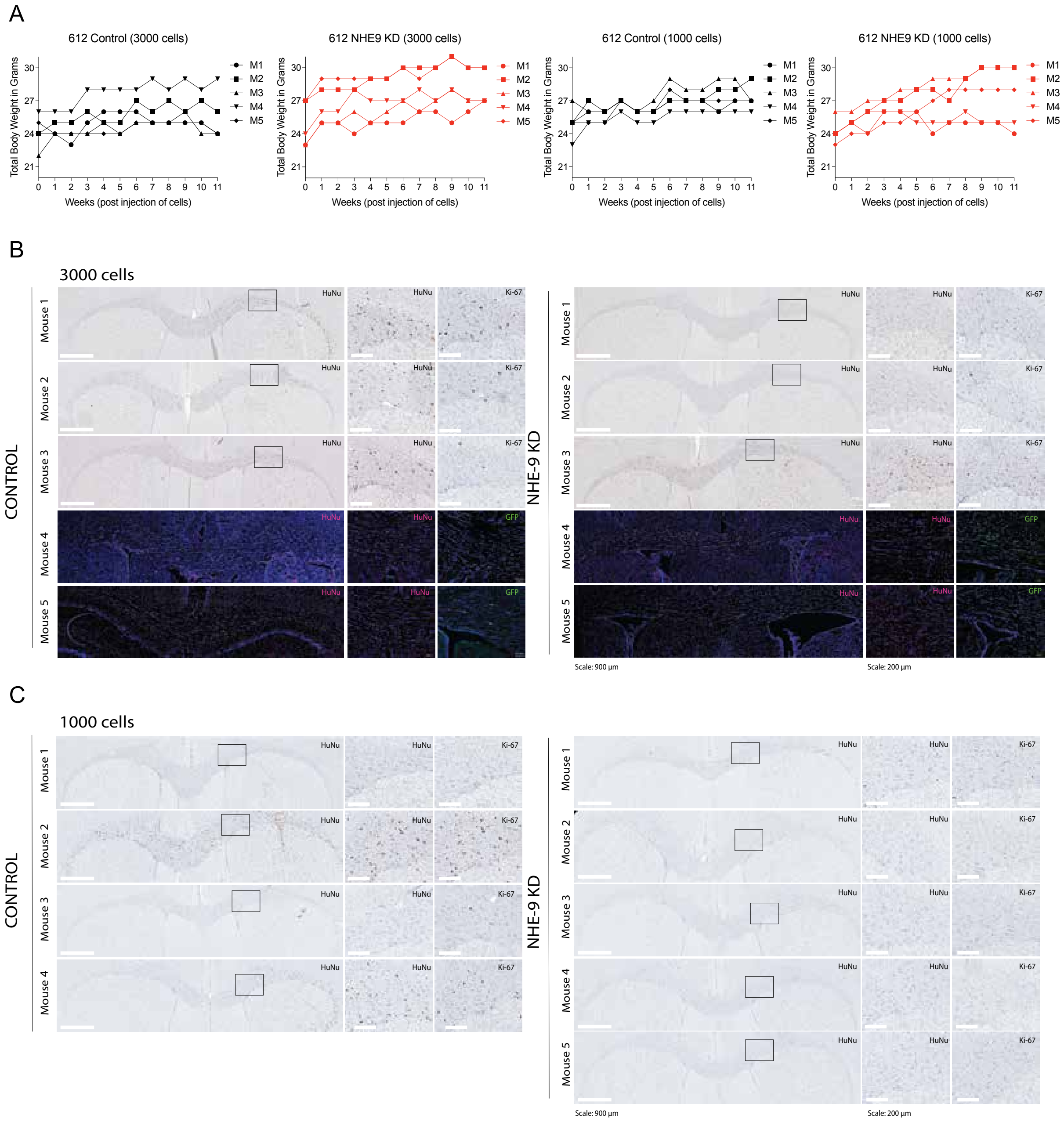

Figure S5: Effect of NHE9 on tumor initiation in mice

Relates to Figure 8.

(A) Total body weight of nude athymic mice injected with GBM 612 control or NHE9 KD cells (3000 or 1000), measured in grams over 11 weeks. (B-C) Immunohistochemistry (IHC) and immunofluorescence (IF) images of sectioned brains from mice injected with GBM 612 cells transduced by lentivirus packaged with empty vector or shNHE9 construct at either 3000 cell dilution (A) or 1000 cell dilution (B). (Scales for IHC, 900 µm or 200 µm; for IF, 50 µm).
