# Supplementary material for: The endosomal pH regulator NHE9 is a driver of stemness in glioblastoma
Source: PNAS Nexus. 2022 Mar 9;1(1):pgac013. doi: 10.1093/pnasnexus/pgac013 (PMC8974362; doi:10.1093/pnasnexus/pgac013)
Supplement: pgac013_Supplemental_Files [file PNASNEXUS-PNASNEXUS-2021-00240-T-s02.pdf › PNASNEXUS-PNASNEXUS-2021-00240-T-s02.pdf]

December 2, 2021

Title: "The Endosomal pH Regulator NHE9 is a Driver of Stemness in Glioblastoma"
Tracking #: 2021-18371
Authors: Ko et al.

Dear Dr. Rao,

Thank you for submitting your manuscript "The Endosomal pH Regulator NHE9 is a Driver of Stemness in Glioblastoma" for consideration by PNAS. We apologize for the delay in returning a decision for your manuscript. After evaluation of its suitability for PNAS, your manuscript was assigned to an editor with expertise in the subject area of your work, who examined the manuscript and identified 3 expert reviewers in the field to assess your work. These reviews, which appear below, were considered by the editor, who determined that the issues raised by reviewers were of sufficient concern to merit declining the opportunity to publish the manuscript in PNAS.

The editor, however, encourages you to submit to *PNAS Nexus*, a gold open access journal from the National Academy of Sciences, with a focus on high-quality, innovative multi-, trans-, and interdisciplinary work across the biological, physical, and social sciences, particularly encompassing engineering and the health sciences.

Because your work was determined to be technically sound and interesting to a broad audience, we believe that the broader portfolio of *PNAS Nexus* would be a better fit. If you choose to revise your work according to the advice of the reviewer(s) and would like to transfer your manuscript for consideration at *PNAS Nexus*, please follow the link below. You will not have to reformat your manuscript for consideration by *PNAS Nexus*.

We hope that the rationale for our decision is clear and that you found the comments of the reviewers helpful. Thank you for choosing PNAS as a potential venue for publishing your work and we hope that you'll consider *PNAS Nexus* for this work and PNAS for future submissions.

Yours,
May R. Berenbaum
Editor-in-Chief

****

Editor's Remarks to Author:

Your article has been reviewed by three experts in the areas of NHE transporters and cell signaling. From the reviews, you will see that your article evoked genuine interest. Nevertheless, two of the reviewers feel that the quality of the work does not warrant publication in a journal with the PNAS standard to publish only ground-breaking, high impact work. As you see, one reviewer is especially concerned that the present work does not substantively advance on your Nat.Com. article from 2005, and after reading both articles myself I must concur with that reviewer. As hinted by the most positive reviewer, reviewer #1, it should be possible in the present day to develop molecular tools to constitutively and selectively manipulate the pH of endosomes and thereby probe the central hypotheses of this article in a definitive fashion. This, certainly, would be the type of quantal progress that is favored for publication in this journal. To summarize, while all of the reviewers, and I myself, find the article of interest, we regret to conclude that the article does not reach the standard required by this journal to support preparation of a revised version. We encourage you to use the reviews to improve the article for another publication venue. Specifically, we point out PNAS Nexus, a new open access research journal launched by PNAS which is also focused on the publication of high-quality, impactful research with broad, interdisciplinary appeal.

The substantive advance in this study is the link between endosomal pH and stemness in glioblastoma, which is both new and significant. To our knowledge, the most precise molecular tool to constitutively and selectively manipulate the pH of endosomes is by regulating NHE9 activity and expression. Indeed, we use molecular approaches of gene silencing, transgenic expression and expression of transport-deficient NHE9 mutants as tools to manipulate recycling endosome pH.

Reviewer Comments:

Reviewer #1:

Suitable Quality?: Yes

Sufficient General Interest?: Yes
Conclusions Justified?: Yes
Clearly Written?: Yes
Procedures Described?: Yes
Supplemental Material Warranted?: Yes

Comments:
GBM is not only the most frequent brain cancer, it is also rapidly fatal. The main reasons for the short survival rates of people with GBM are the presence of cancer stem cells and limited therapeutic modalities. Thus, identifying underlying mechanisms and new therapeutic targets is important. This manuscript very strongly makes the case for NHE9 being a targetable mechanism and the data presented are very strong. The manuscript itself was extremely well-written and below are some comments for consideration.

Thank you for your positive evaluation!

Based on their findings and the findings of others that deacidification of endosomes and lysosomes increases the chemotherapeutic actions of temozolomide (TMZ) it would have been interesting to know how the cells expressing high and low levels of NHE9 respond to TMZ.

Thank you for your interest! We have indeed observed that NHE9 substantively increases IC_{50} for temozolomide in GBM cells *in vitro*. Although exciting and significant, these data are not included in the manuscript as more work will need to be done to validate our findings (e.g., *in vivo* tests) which is out of the scope for this study.

Because endosome pH is central to this story it is not clear why they chose to include such data as a supplement. The pH data should be more prominently displayed.

We agree that pH is important as an underlying mechanism to our observations. However, we have previously reported on pH calibration and measurement in GBM cells. Therefore, the pH measurement data has been placed in the supplement, while the new stemness phenotypes and effect of pH modulators are given prominence in the main figures.

If pH is of such central importance than what happens when endosomes are acidified. Several approaches could be used to acidify endosomes.

This is an excellent perspective. Indeed, the effects of endosome acidification have been studied by us and several other groups and reported in the literature (PMID: 32107410; PMID: 29946028; PMID: 24035762; PMID: 24065030). Loss of function mutations in endosomal NHE are linked to autism and other neurodegenerative and neurodevelopmental disorders. Briefly, acidification resulting from loss of endosomal NHE activity is associated with diminished expression of cell surface receptors and transporters, and is restored upon pH correction. Thus, acidification and alkalinization of the endosome have reciprocal effects on membrane protein retention and stability, providing additional confidence in our current findings.

They cited a manuscript (ref #20) that reported that no change in cytosolic pH was observed in response to manipulation of NHE9 expression. They should comment on why they think that this is a repeatable finding, possibly indication that the pool of hydrogen ions is too small in endosomes to make a difference in cytoplasmic pH. They should make clear that they are referring to cytoplasmic or cytosolic.

This is correct: endosomal volume is small relative to the cytoplasm, and there are efficient mechanisms at the plasma membrane (chiefly NHE1) to regulate cytosolic pH. Similar findings have been reported in the literature (PMID: 21881004). We have included this additional reference and changed wording to cytosolic pH throughout the manuscript.

What affects, if any, were observed in endosome morphology or intracellular distribution patterns because of NHE9 expression levels and pH?

There were no morphological or distribution changes apparent, although we have not investigated these in rigorous detail.

Reviewer #2:

Suitable Quality?: No
Sufficient General Interest?: No
Conclusions Justified?: No
Clearly Written?: Yes

Procedures Described?: Yes
Supplemental Material Warranted?: Yes

Comments:
Ko et al. studied the role of the endosomal sodium/hydrogen exchanger NHE9 in glioblastoma, a very aggressive form of brain cancer. Based on their findings, the authors propose that NHE9 contributes to oncogenic signalling in glioblastoma cells by stabilizing receptor kinases at the plasma membrane and preventing them from degradation.

The manuscript is well written. The experiments are well described and follow standard protocols in the field. Experiments were carefully executed.

We thank you for your encouraging comments!

My major concern for this work is the significant overlap of reported findings with a previous study published by the group.

1. Overlap with previous study (Kondapalli KC et al, Nat Commun. 2015 Feb 9;6:6289. doi: 10.1038/ncomms7289): New findings reported are incremental; very similar results have been published by the group already. The same patient-derived stable patient-derived cell lines (GBM 276 and GBM 612) with NHE9 knock-down or overexpression were used. Very similar in vitro and in vivo studies with these cell lines have already been reported. In the current manuscript, the initial observation for a single receptor tyrosine kinase (EGFR) has been extended to additional oncogenic receptor tyrosine kinases at the cell surface. Yet, the basic mechanisms proposed are the same (lack of internalization and degradation).

We regret making the novelty of our work unclear. To clarify, the **link between endosomal pH and stemness is both new and significant**. New mechanistic insights focus on the role of JAK/STAT signaling and induction of pluripotent factors Oct4 and Nanog that mediate the effect of NHE9 on stemness properties in GBM cells. To be sure, as a starting point, we have confirmed and extended our previous findings on receptor tyrosine kinase stability using our previously validated GBM cell lines. To emphasize this point, we have added the following to the Results: "*These observations confirm and extend our earlier findings on EGFR persistence in GBM (20), and serve as a necessary starting point to determine the downstream oncogenic consequences of NHE9 expression".*

2. I feel conclusions drawn are premature. I am worried about clonal selection artifacts, the same cell lines with stable NHE9 knock-down or overexpression are used throughout (as in the previous manuscript). The authors report a nice series of patient-derived cell lines encompassing a large range of NHE9 expression (Fig. 1 B). These cell lines should be used for in vitro and in vivo studies. If indeed NHE9 plays a major role in oncogenic signalling in glioblastoma, there should be clear differences between the low and high NHE9 expressing cell lines.

Our concern with the reviewer's suggestion is that the patient derived GBM cells shown in Fig. 1B are non-isogenic and derived from different GBM molecular subtypes. Therefore, a simple correlation between NHE9 levels and oncogenic signaling across all cell lines is not guaranteed and should not be considered as a definitive test of our hypothesis. A more rigorous approach is to evaluate the effect of NHE9 knockdown and expression in isogenic sets, which we have done. In principle, this approach could be extended to many more cell lines in future studies. We cite the following observation that should convince the reviewer of the robustness of our findings: endosomal acidification has the reciprocal effect on membrane receptor stability, and has been demonstrated in a range cell models (e.g., primary astrocytes, neurons, HeLa, HEK293) by our group and others (PMID: 32107410; PMID: 29946028; PMID: 24035762; PMID: 24065030).

3. The reported correlation of NHE9 expression with pSTAT3 is weak - questioning the extendability of the findings obtained with the two selected cell lines. The same applies for other correlations reported (JAK2, Suppl. Fig. 2A).

We have deleted the supplemental data. We note that although important, JAK/STAT is one of many oncogenic signaling pathways in GBM. Therefore, the modest correlation between NHE9 and pSTAT or JAK2 across a large number of diverse GBM subtypes is not unexpected. Despite the relatively weak correlation, the significance of the correlation is high (p<0.0001; see figure), and this led us to evaluate the pathway in biochemical detail.

Expression correlation TCGA NHE9-STAT3
NHE9-JAK2

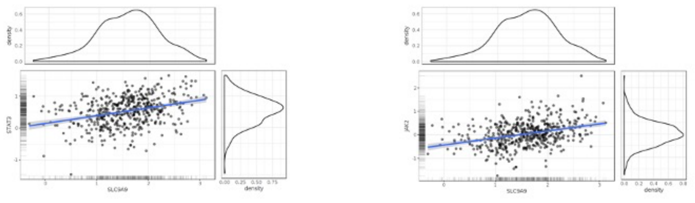

Pearson r	
r	0.3302
95% confidence interval	0.2488 to 0.4069
R squared	0.1090
P value	
P (two-tailed)	<0.0001
P value summary	****
Significant? (alpha = 0.05)	Yes
Number of XY Pairs	489

Pearson r	
r	0.3458
95% confidence interval	0.2652 to 0.4215
R squared	0.1196
P value	
P (two-tailed)	<0.0001
P value summary	****
Significant? (alpha = 0.05)	Yes
Number of XY Pairs	489

4. Use of non-specific inhibitors: monensin and bafilomycin are highly unspecific and will affect luminal pH in many intracellular organelles. Therefore, results obtained with these compounds cannot be simply extended to endosomal pH.

We regret that we did not clarify that our strategy includes **both specific and non-specific approaches** to query the role of endosomal pH in GBM cells. Specific approaches include the molecular manipulation of NHE9 levels and the use of transport inactive NHE9 mutants, which change endosomal pH in a predictable, quantifiable and stable way. We have now added the following caveat to the Results: *"we used pharmacological approaches to alkalinize the endosomal lumen and bypass the requirement for NHE9, with the caveat that these agents are not compartment-specific".*

Reviewer #3:

Suitable Quality?: No
Sufficient General Interest?: Yes
Conclusions Justified?: Yes
Clearly Written?: Yes
Procedures Described?: No
Supplemental Material Warranted?: Yes

Statistical or Methodological Comments:
1. It is stated that most of the Western blot experiments were 'biological triplicates'. It is not clear whether this means triplicate blots of the same protein lysates or three independent experiments.

We have replaced the term biological replicates with the term "independently transfected cell cultures" throughout the manuscript.

2. There is no indication in the figure legends or the methods as to whether the data represent the mean {plus minus} SEM or SD. The data points should be shown on the bar graphs and the whiskers should preferably show the SD to better indicate the dispersion of the data from the mean.

We have clarified methods in the section on statistical analysis.

Comments:
Summary:
The study by Ko and co-workers extends previous work from this laboratory which reported that expression of the endosomal pH-regulating Na+/H+ exchanger isoform 9 (NHE9) is upregulated in glioblastoma (GBM) cells, leading to aberrant alkalinization of the recycling endosomal compartment. This in turn was found to misdirect internalized epidermal growth factor receptors (EGFRs) along the recycling endosomal rather than degradative pathway, resulting in elevated plasma membrane levels and prolonged signaling from EGFRs, a contributing factor in malignancy of this brain tumor type. In the present study, the authors examined the hypothesis that dysregulation of pH in recycling endosomes in GBM cells due to increased NHE9 expression, rather than being receptor selective, instead indiscriminately elevates surface expression of other receptor tyrosine kinases (RTK), leading to protracted broad-based oncogenic signaling. To this end, the authors screened for NHE9 expression in a diverse panel of 10 patient-derived GBM cell lines established from samples obtained at Johns Hopkins Hospital. The analysis indicated that GBM cell lines with high NHE9 expression indeed correlated with elevated expression of other RTKs (insulin-like growth factor receptor 1 (IGF-1R), insulin receptor (IR), and platelet-derived growth factor receptor alpha (PDGFRα)). These changes correlated with elevated JAK2-STAT3 signaling and selective induction of stem cell pluripotency genes Oct4 and Nanog which repress glial differentiation. The significance of these changes was examined using over-expression and shRNA knock-down approaches in vitro and tumor initiation in vivo.

Comments:
This is a thought-provoking study that builds on original observations from this laboratory about the importance of endosomal pH homeostasis and its relevance to cancer biology, and in particular to glioblastomas. The manuscript is well written and, on the whole, provides a compelling case for abnormal NHE9 expression as an upstream driver of RTK oncogenic signaling and as a potential target for drug therapy which should be of broad interest to the cancer field. That said, there are a number of questions and concerns about aspects of the data that need to be addressed to raise confidence in the final results and their interpretation.

Thank you for your careful and thorough review!

1. This study makes extensive use of antibodies, yet there is not a single mention in the Materials and Methods section of the source and veracity of each of these antibodies. This needs to be carefully documented as many of the blots show only thin sections of the blots highlighting the presumed targets, leaving it difficult to ascertain their specificity.

Details on antibody source have been added to Methods.

2. It is stated that most of the Western blot experiments were 'biological triplicates'. It is not clear whether this means triplicate blots of the same protein lysates or three independent experiments.

Biological replicates refer to independent experiments. We have clarified this in the section on statistical analysis.

3. There is no indication in the figure legends or the methods as to whether the data represent the mean {plus minus} SEM or SD. The data points should be shown on the bar graphs and the whiskers should preferably show the SD to better indicate the dispersion of the data from the mean.

We have clarified this in the section on statistical analysis.

4. While NHE9 mRNA levels differ significantly between GBM-276 (low expression) and GBM-612 (high expression), the difference in the endogenous expression of NHE9 protein between these two cell lines was not clearly defined. Fig. S1B-C shows expression of NHE9 (73 kDa) in GBM-612 cells, but not GBM-276. Also, the size of the NHE9-GFP band in Fig. S1C (GFP should increase NHE9 weight by an additional 27 kDa) seems unexpectedly high (i.e., 225 kDa). Please explain the unaccounted size difference.

Unfortunately, available antibodies against NHE9 are not of sufficient quality to detect low levels of endogenous protein. NHE proteins have been repeatedly observed to run aberrantly on SDS-PAGE, and higher order bands typically consist of dimers.

5. In Fig. S1D, the patient screen identified RTKs IGF-1R, IR and PDGFR-A, but what about expression of EGFR? Was it not detected in the screen as identified in earlier studies?

The difference in p-EGFR was not statistically significant, possibly due to technical issues with the dot blot (for example, the EGFR sample was located at the edge of the blot). False negatives and positives are not unexpected with this experimental approach, and therefore we used the blot as a tool for discovery and followed up with systematic validation as described.

6. The authors indicate that endosomal pH was lower in GBM-276 compared to GBM-612, consistent with a role for NHE9 in limiting lumen acidification. What about expression of other transporters (Cl-/H+ exchangers of the CLC family) and H+ pumps (V-ATPase) that are also responsible for endosomal pH - was this checked and, if, so, did their expression change?

It is possible that expression of many transporters that influence pH is altered in GBM 276 and we have not conducted a systematic analysis of this. The key observation is that transgenic expression of NHE9 in GBM 276 elevated endosomal pH to comparable levels with GBM 612. We used this as a tool to query the effect of endosomal pH on stemness.

7. In Fig. 1D and 1 E, why are the p-IR and p-IGF-1R signals together (cannot be distinguished) on the same blot and not on separate blots?

The antibody that we used in Fig. 1D and 1H (cell signaling Rabbit mAb #3024) detects both p-IGF1R and p-IR. The antibody was produced by immunizing animals with a synthetic phospho-peptide corresponding to residues surrounding Tyr1135/1136 of human IGF-1receptor beta and detects p-IR as well. However, it does not cross-react with other receptor tyrosine kinases.

In Fig. 3H we use multiple different antibodies that detect p-IGF1R show robust and specific increase in p-IGF1R signal with ectopic NHE9.

8. The inset boxes in Fig. 2C are completely misaligned with the intended highlighted cell.

This has been corrected.

9. Fig 3D, the tubulin signals appears of lower quality compared to other blots. This blot also seems identical to the tubulin blot in Fig. 4E. Please clarify.

Although the tubulin signals in Fig 3D are light, they are of good quality for densitometry. We have now clarified in the legend to Fig. 4E that different sections of the same gel are shown in the two panels, hence the loading control is the same. Similarly, we have noted that panels 4I and 4K represent different sections of the same gel.

10. Fig. 3E, is the p-STAT3 normalized to STAT3 and/or tubulin? Why are there 2 bands for p-STAT3 in panel F but not so evident in

panel D?

p-STAT3 was normalized to tubulin. Multiple phosphorylated bands are seen on both panels but appear more prominent in panel F, likely due to differences in exposure and/or relative phosphorylation.

11. Fig. 3H-J, the figure indicates a 25-h time point, but the text states 24-h.

The text has been corrected to 25 h.

12. Fig. S2B, indicates use of S31-201 or stattic, yet legend states cryptotanshinone or stattic used. Also, why is Nanog mRNA expression inhibited by S31-201, but not stattic? The first lane of Nanog blot is not very clear. Need better blot.

We have corrected the legend to Fig. S2B. Nanog is partially inhibited by Stattic in the Western blot shown (note that the +NHE9 condition is not elevated above vector control as it should be in the absence of STAT inhibitors). We note that the lowest concentration of Stattic from the dose response in panel D was used. Although some cosmetic edge lane artifact appears, it does not detract from the conclusion.

13. Fig. 3K. How was the relative mRNA expression normalized, as the control levels (GBM-276 GFP) vary and are not uniformly set to 1?

This has been corrected.

14. Fig. 6. Monensin and bafilomycin were used to alkalinize the endosomal compartment. However, it should be emphasized in the text that these compounds will also alkalinize other acidic compartments, such as the Golgi and secretory vesicles, so difficult to attribute their effects solely to effects on endo-lysosomes.

We used both specific and non-specific approaches to query the role of endosomal pH in GBM cells. Specific approaches include the manipulation of NHE9 levels and the use of transport inactive NHE9 mutants. We have now added the following caveat to the Results: “we used pharmacological approaches to alkalinize the endosomal lumen and bypass the requirement for NHE9, with the caveat that these agents are not compartment-specific”.

15. In the Discussion, there is brief mention of the involvement of aberrant V-ATPase expression/activity in malignancy of glioblastoma. The authors state that V-ATPase inhibitors that alkalinize endo-lysosomes are being explored as therapeutic adjuvant therapy to inhibit GBM. This approach would seem contrary to the authors' supposition that development of NHE9 antagonists that would acidify endosomes might be a viable treatment strategy for GBM. Additional discussion of these contrary approaches seems warranted.

We have discussed this in some detail. Specifically, the effect of pH is compartment-specific. V-ATPase activity in the early endosomes is oncogenic in GBM and presents a viable treatment strategy. We have added the italicized section to the following discussion: “However, tumorigenic alterations in pH appear to be compartment-specific: in a model of breast cancer, decreased expression of NHE6 correlated with hyper-acidification of early endosomes resulting in drug sequestration and chemotherapeutic resistance (21). *Consistent with the oncogenic role of acidic pH in early endosomes, increased V-ATPase activity in this compartment leads to increased shedding of oncogenic extracellular vesicles (64).* On the other hand, elevated expression of the trans-Golgi network isoform NHE7 in a pancreatic cancer model also resulted in hyperacidification of compartmental lumen and activation of plasma membrane Na^+/H^+ exchange (22), mimicking NHE1-mediated regulation of cytoplasmic pH and extracellular acidification common to a wide range of cancer types.”
